# Synthesis of an Anti-CD7 Recombinant Immunotoxin Based on PE24 in CHO and *E. coli* Cell-Free Systems

**DOI:** 10.3390/ijms232213697

**Published:** 2022-11-08

**Authors:** Simon K. Krebs, Marlitt Stech, Felix Jorde, Nathanaël Rakotoarinoro, Franziska Ramm, Sophie Marinoff, Sven Bahrke, Antje Danielczyk, Doreen A. Wüstenhagen, Stefan Kubick

**Affiliations:** 1 Branch Bioanalytics and Bioprocesses (IZI-BB), Fraunhofer Institute for Cell Therapy and Immunology (IZI), Am Mühlenberg 13, 14476 Potsdam, Germany; 2 Institute for Biotechnology, Technical University of Berlin, Ackerstrasse 76, 13355 Berlin, Germany; 3 Institute of Pharmacy, Freie Universität Berlin, Königin-Luise-Strasse 2 + 4, 14195 Berlin, Germany; 4 Institute of Chemistry and Biochemistry, Freie Universität Berlin, Takustrasse 6, 14195 Berlin, Germany; 5 Glycotope GmbH, Robert-Roessle-Strasse 10, 13125 Berlin, Germany; 6 Faculty of Health Sciences, Joint Faculty of the Brandenburg University of Technology Cottbus-Senftenberg, The Brandenburg Medical School Theodor Fontane and the University of Potsdam, 14476 Potsdam, Germany

**Keywords:** cell-free protein synthesis, CFPS, cell-free expression, CFE, RIT, Pseudomonas Exotoxin A, GroEL/GroES, DnaK/DnaJ/GrpE, trigger factor

## Abstract

Recombinant immunotoxins (RITs) are an effective class of agents for targeted therapy in cancer treatment. In this article, we demonstrate the straight-forward production and testing of an anti-CD7 RIT based on PE24 in a prokaryotic and a eukaryotic cell-free system. The prokaryotic cell-free system was derived from *Escherichia coli* BL21 Star^TM^ (DE3) cells transformed with a plasmid encoding the chaperones groEL/groES. The eukaryotic cell-free system was prepared from Chinese hamster ovary (CHO) cells that leave intact endoplasmic reticulum-derived microsomes in the cell-free reaction mix from which the RIT was extracted. The investigated RIT was built by fusing an anti-CD7 single-chain variable fragment (scFv) with the toxin domain PE24, a shortened variant of Pseudomonas Exotoxin A. The RIT was produced in both cell-free systems and tested for antigen binding against CD7 and cell killing on CD7-positive Jurkat, HSB-2, and ALL-SIL cells. CD7-positive cells were effectively killed by the anti-CD7 scFv-PE24 RIT with an IC_50_ value of 15 pM to 40 pM for CHO and 42 pM to 156 pM for *E. coli* cell-free-produced RIT. CD7-negative Raji cells were unaffected by the RIT. Toxin and antibody domain alone did not show cytotoxic effects on either CD7-positive or CD7-negative cells. To our knowledge, this report describes the production of an active RIT in *E. coli* and CHO cell-free systems for the first time. We provide the proof-of-concept that cell-free protein synthesis allows for on-demand testing of antibody–toxin conjugate activity in a time-efficient workflow without cell lysis or purification required.

## 1. Introduction

Cancer kills millions of people each year despite impressive achievements in oncology during the last 100 years. Currently, most cancer cases are initially treated by classic chemo- and radiotherapy regimens. More recently, personalized targeted therapy is increasingly being used as second-line and third-line treatment options and, for some indications, emerged as a first-line treatment. Examples for targeted therapy first-line drugs include anti-Her2/neu monoclonal antibody Trastuzumab, BRAF kinase inhibitor Vemurafenib, and anti-IL2 immunotoxin tagraxofusp. Targeted cancer therapy is potentially superior to systemically effective cytotoxic drugs due to off-target effects and the concurrent narrow therapeutic window of undirected anti-cancer cytotoxins. Survival rates of most malignancies improved drastically over the last 100 years due to new drugs and improved regimens but many types of cancer are still highly lethal and cannot be cured with the drugs approved for clinical use. This demonstrates the need for further treatment options [1,2,3,4].

A promising class of targeted anti-cancer therapeutics are antibody–toxin conjugates, such as antibody–drug conjugates (ADCs) and recombinant immunotoxins (RITs). They target a cancer-associated antigen and the attached toxin moiety specifically eradicates malignant cells, while healthy cells remain unaffected. Thereby, the therapeutic window is enlarged [5,6]. In contrast to ADCs, the cytotoxic compound in RITs is a protein, which in most cases is of non-human origin. Prominent candidates for toxin domains include recombinant variants of the bacterial toxins Pseudomonas Exotoxin A (PE) and diphtheria toxin (DT), which both target eukaryotic elongation factor 2 (eEF2). Furthermore, plant-derived toxins, such as Ricin and Gelonin, are frequently used, which both target eukaryotic 28S ribosomal RNA [7]. The associated cell killing triggered by translation inhibition and according downstream effectors therefore exclusively affects eukaryotic cells [8]. Accordingly, RIT production with toxin domains, which target the eukaryotic translation process, is challenging in eukaryotic expression hosts due to self-intoxication effects [9,10]. To avoid self-intoxication, prokaryotic expression hosts, such as *Escherichia coli,* are utilized. The RITs are then usually purified from inclusion bodies (IBs) [11], which represents a time-intensive procedure with relatively low efficiency [12,13]. In fact, the three FDA approved RITs, denileukin diftitox (DT-IL-2, DAB389IL-2; Ontak^®^), tagraxofusp-erzs (DT-IL-3, SL-401, Elzonris^®^), and moxetumomab pasudotox (anti-CD22 dsFv-PE38, CAT-8015 or HA22, Lumoxiti^®^), are produced in *E. coli* cells and then refolded and purified from IBs [14,15,16,17].

Immunotoxins suffer from several drawbacks that need to be overcome before fully unfolding their clinical potential. These critical issues concern all domains of the RIT, namely targeting domain, linker domain, and toxin domain. For example, the immunogenicity of non-human antibodies and toxins leads to respective neutralizing antibodies that limit the RIT’s efficacy. Furthermore, several potentially life-threatening conditions evoked by off-target or on-target effects were observed in patients treated with RITs, such as capillary leak syndrome, cytokine release syndrome, hepatotoxicity, nephrotoxicity, and cardiac toxicity [17,18]. To overcome these issues and gain more effective RITs, iterative redesign of the RIT coding sequence has to be undertaken, such as humanization of the antibody, affinity maturation of complementarity determining regions, conversion to a bispecific format, improvement of linker stability, linker functionality, and/or removal of B and T cell epitopes of the toxin domain [8,17,19,20,21,22,23]. We anticipate that cell-free protein synthesis (CFPS) may be applied as an economic and time-efficient tool to evaluate the impact of sequence redesign on RIT efficacy during its development process. CFPS enables on-demand production of proteins of interest (POIs) within a few hours, using the protein translation machinery of disintegrated cells. The option for massive parallelization using linear expression templates and the open nature of cell-free systems allows economic, flexible, high-throughput screening of POIs on an analytical scale without the need to create genetically modified organisms or to perform cell lysis of the expression host or purification of the POI [24,25].

In this study, we explored *E. coli* and Chinese hamster ovary (CHO)-based CFPS as a tool for straight-forward RIT production and in vitro testing for antigen binding and cytotoxicity. We aimed to streamline the RIT development process by avoiding cumbersome IB solubilization and purification to allow flexible on-demand synthesis of RITs. The RIT chosen for this proof-of-concept targets CD7 by a single-chain variable fragment (scFv) domain, which was linked to PE24 by a flexible GS linker. The toxin domain PE24 (also known as PE[LR]) has been developed by Pastan and colleagues [26]. Compared to full-length PE (also known as PE66), in PE24, the native CD91 receptor binding domain Ia and the translocation domain II, except the furin cleavage site, are deleted. PE24 is the shortest version of PE66 currently known to effectively kill target cells when equipped with an appropriate binding domain [27]. CD7, the RIT’s target, is a 40 kDa glycosylated membrane protein mainly expressed on healthy T and NK cells including their progenitors and is involved in T cell activation and immune cell interaction [28]. Several lymphatic diseases are associated with CD7-positive T cells or B cells, such as most cases of T cell acute lymphoblastic leukemia (T-ALL) [29], some cases of acute myeloid leukemia (AML) [30], and acute B lymphoblastic leukemia (B-ALL) [31], as well as graft vs. host disease (GvHD) [32,33]. Promising treatment options have recently been approved by the FDA for B-ALL and AML [34,35]. Potential indications for an anti-CD7 RIT include relapsed or refractory T-ALL [36,37] and steroid-refractory acute GvHD [38,39] as well as other treatments that require T cell depletion, such as allogeneic hematopoietic stem cell transplantation (HSCT) [40,41]. However, CD7-targeted T cell depletion renders the patient immune-deficient and therefore is associated with great risks for opportunistic infections during treatment [37].

CHO cells are the most commonly used expression hosts for production of therapeutic proteins, particularly for therapeutic antibodies [42]. This is mainly due to their ability for complex post-translational modifications, such as glycosylation, and their recognition as a safe expression host by the FDA [43]. However, due to self-intoxication, CHO cells are not a viable option for production of RITs, whose toxin domain targets eukaryotic translation. Therefore, to avoid work-intensive IB solubilization and purification associated with *E. coli* cell-based expression, an *E. coli* cell-free system was chosen for RIT production. It is considered the most productive cell-free system [44]. In order to increase the soluble yield of the RIT, the *E. coli* cell-free system was supplemented with the chaperones GroEL/GroES (ELS), DnaK/DnaJ/GrpE (KJE), or Trigger Factor (TF) in different combinations. Chaperones are indispensable, evolutionary, conserved folding helpers required to maintain protein homeostasis in cells [45,46]. Supplementation of chaperones was found to often increase soluble yields and activity of the POI by assisting correct folding of the heterologously expressed POI in *E. coli* cell-based and in *E. coli* cell-free expression [47,48].

Aiming to provide a solution for endotoxin-free RIT production in a eukaryotic system, we further evaluated CHO-based CFPS for RIT production. The CHO cell-free system used here contains endoplasmic reticulum (ER)-derived microsomes. We therefore speculated that CHO-based CFPS might be less susceptible to PE-mediated translation inhibition than eukaryotic cell-based production. Microsomes are vesicular structures to which cell-free synthesized proteins can be co-translationally targeted, e.g., by the signal peptide from the bee toxin melittin [49]. Microsome-translocated proteins are physically separated from the translation apparatus by the lipid bilayer of the microsomes. Accordingly, eEF2 would be protected from PE24-mediated ribosylation. Importantly, microsome-extracted proteins most often show higher activity than proteins that have not been translocated. This is likely due to an environment that is beneficial for correct folding, such as disulfide bond promoting redox potential and ER-residing chaperones [50,51]. Furthermore, post-translational modifications, such as core glycosylation and disulfide bond formation, can be achieved in microsomes [49,50,52].

To our knowledge, neither CHO nor *E. coli* CFPS have ever been evaluated for RIT production. Here, we demonstrate the proof-of-concept for RIT in vitro testing using a CHO-based cell-free system containing ER-derived microsomes and an *E. coli*-based cell-free system supplemented with ELS using an anti-CD7 scFv-PE24 RIT as a model. Using cell-free systems for on-demand synthesis of RITs might help to streamline RIT research and development.

## 2. Results

### 2.1. E. coli-Based Cell-Free Production of PE24 RIT

**Figure 1:**
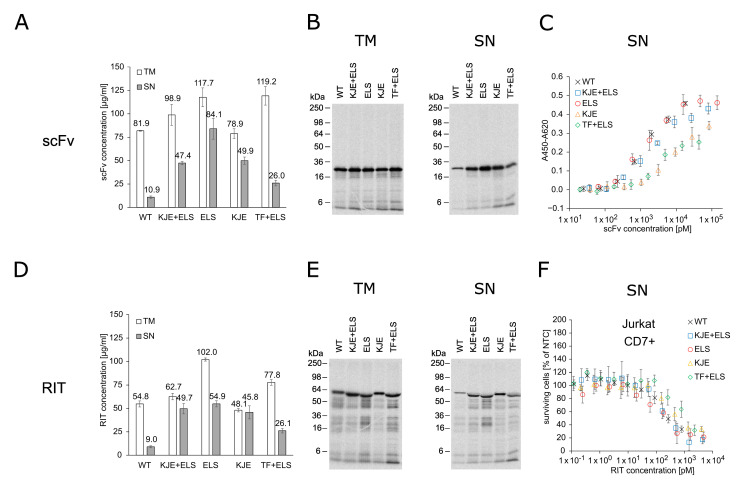
Quantification, autoradiography, antigen binding, and cytotoxicity of *E. coli* cell-free-produced proteins with and without chaperones. (**A**) Liquid scintillation counting for quantification of scFv in TM and SN fraction. Error bars represent the standard deviation of triplicate analysis. (**B**) Autoradiography of reducing denatured 12% Tris-Glycine SDS-PAGE gel of scFv (29.9 kDa) in TM and SN fraction. (**C**) Indirect ELISA to analyze binding of the scFv to its target antigen CD7. Absorbance values in wells without CD7 were subtracted from absorbance values in wells coated with CD7. Error bars represent the standard deviation of triplicate analysis. (**D**) Liquid scintillation counting for quantification of RIT in TM and SN fraction. Error bars represent the standard deviation of triplicate analysis. (**E**) Autoradiography of reducing denatured 12% Tris-Glycine SDS-PAGE of RIT (56.4 kDa) in TM and SN fraction. (**F**) MTT assay of RIT on CD7-positive Jurkat cells. Absorbance values of samples were normalized in respect to absorbance of no-template control (NTC), which was identically diluted and treated as the sample. Error bars represent the standard deviation of normalized cell survival performed in triplicate. Abbreviations: scFv: single-chain variable fragment; RIT: recombinant immunotoxin; TM: translation mix; SN: supernatant; WT: wildtype BL21 StarTM (DE3) w/o chaperones; KJE+ELS: WT transformed with plasmid coding for DnaK/DnaJ/GrpE and GroEL/GroES; ELS: WT transformed with plasmid coding for GroEL/GroES; KJE: WT transformed with plasmid coding for DnaK/DnaJ/GrpE; TF+ELS: WT transformed with plasmid coding for Trigger Factor and GroEL/GroES.

RITs are commonly produced using *E. coli* cell-based expression with the subsequent purification and refolding of IBs [17]. Using a productive, well-established *E. coli* expression strain as a basis for a cell-free system, we seek practical, economic, and time-saving advantages when using CFPS for RIT screening purposes instead of *E. coli* cell-based production. In this study, we evaluated an *E. coli*-based cell-free system for the production and testing of the anti-CD7 model RIT. The *E. coli* cell-free system was equipped with chaperones ELS, KJE, and TF [47] in different combinations by transformation of the *E. coli* strain BL21 Star^TM^ (DE3) with the corresponding plasmid prior to lysate preparation. Soluble yield and functionality of the scFv and the scFv-PE24 RIT synthesized in presence and absence of chaperones were compared in order to identify the most suitable chaperone for *E. coli* cell-free RIT production. Liquid scintillation counting revealed that, for the cell-free system derived from the *E. coli* strain without chaperones, less than 20% of the total protein (translation mix, TM) was found in the soluble fraction (supernatant, SN). In contrast, the presence of chaperones substantially produced 2.5-fold to 8-fold increased yields in the soluble fractions (Figure 1A,D). For scFv synthesis, the highest soluble yield was found for the cell-free system prepared from the strain transformed with the plasmid coding for ELS. For RIT synthesis, cell-free systems supplemented with KJE+ELS, ELS, or KJE equally performed regarding yield in the soluble fraction, while the lowest increase in soluble yield was found for the cell-free system equipped with TF+ELS. Visualization of the synthesized proteins by autoradiography showed distinct signals at the expected molecular weight. In addition, weaker signals at lower molecular weights were detected (Figure 1B,E). Faint signals were furthermore detected in the autoradiograph of the no-template control (NTC), indicating minor amounts of residual endogenous mRNA present in the lysate (Figure S1A). In the corresponding blue gels, the overexpressed chaperones could be detected in contrast to BL21 Star^TM^ (DE3) wildtype without chaperones (Figure S1B–D). To assess the impact of the chaperones on cell-free-produced scFv functionality, an indirect eight-point enzyme-linked immunosorbent assay (ELISA) against CD7 with unpurified SN was performed (Figure 1C). The IC_50_ values of the scFv synthesized in the different *E. coli* cell-free systems were in a similar range within one log. IC_50_ values determined were 1.40 nM for wildtype BL21 Star^TM^ (DE3), 1.91 nM for KJE+ELS, 1.24 nM for ELS, 8.38 nM for KJE, and 2.76 nM for TF+ELS. As expected, the NTC did not demonstrate binding (Figure S2). Furthermore, to determine the chaperones’ influence on the cytotoxicity of the RIT, an 3-(4,5-dimethylthiazol-2-yl)-2,5-diphenyltetrazolium bromide tetrazolium (MTT) assay on CD7-positive Jurkat cells was performed (Figure 1F). For each sample and the corresponding NTC, a 10-point dilution series was directly prepared from unpurified SN. The absorbance value of the dilution of each sample was normalized for the absorbance of the NTC value at the respective dilution, giving the percentage of surviving cells in relation to NTC-treated cells. This allowed us to mathematically compensate for growth inhibitory effects evoked by the addition of highly concentrated unpurified SN fraction to cells (Figure S3A,B). The MTT assay revealed comparable IC_50_ values with 176 pM for BL21 without chaperones, 172 pM for KJE+ELS, 139 pM for ELS, 310 pM for KJE, and 901 pM for TF+ELS. We noted that ELS produced the highest soluble yield and slightly increased activity for scFv and RIT compared to the other *E. coli* cell-free systems. Based on the data, we decided to proceed for the following experiments with the *E. coli* cell-free system equipped with ELS.

**Figure 2:**
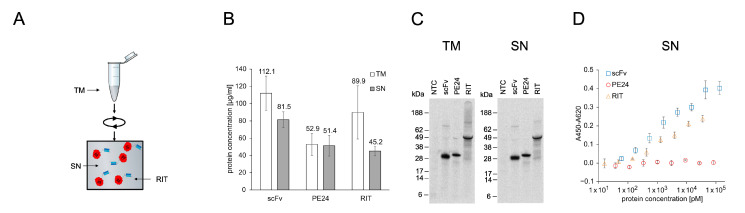
Quantification, autoradiography, and antigen binding of *E. coli* cell-free-produced proteins with GroEL/GroES chaperones. (**A**) Schematics of *E. coli* cell-free protein synthesis. The RIT was produced in reaction vessels containing the translation mix (TM). After synthesis, the TM was centrifuged and the supernatant (SN) containing soluble protein was used for downstream assays. (**B**) Liquid scintillation counting for quantification of proteins in TM and SN fraction. Error bars represent the standard deviation of four independent experiments each performed in triplicate. (**C**) Autoradiograph of reducing denatured 10% Bis-Tris SDS-PAGE. Expected molecular weights are 29.9 kDa for scFv, 28.9 kDa for PE24, and 56.4 kDa for RIT. (**D**) Indirect ELISA of proteins against CD7. Absorbance values in wells without antigen were subtracted from absorbance values in wells coated with CD7. Error bars represent the standard deviation of triplicate analysis. Abbreviations: scFv: single-chain variable fragment; PE24: 24 kDa variant of Pseudomonas Exotoxin A; RIT: recombinant immunotoxin; TM: translation mix; SN: supernatant.

The scFv, PE24, and the RIT were synthesized in the *E. coli* cell-free system with ELS chaperones and the soluble fraction (SN) was prepared (Figure 2A). Liquid scintillation counting determined the yield of soluble protein to be between 45 and 82 µg/mL (Figure 2B), which was comparable to scFv and RIT yield obtained for the ELS supplemented cell-free system in the chaperone screening (Figure 1A,D). The POIs showed their expected molecular weight in the autoradiograph and weaker signals at lower molecular weights (Figure 2C). In an indirect 8-point anti-CD7 ELISA with unpurified SN, an IC_50_ value of 1.07 nM was calculated for the scFv binding to CD7 (Figure 2D). The determined IC_50_ value was in positive agreement with the data for ELS-supplemented CFPS in the chaperone screening (Figure 1C). The RIT bound to CD7 with an IC_50_ value of 2.01 nM, while for PE24, no binding was detected. The NTC did not show any binding either (Figure S4).

**Figure 3:**
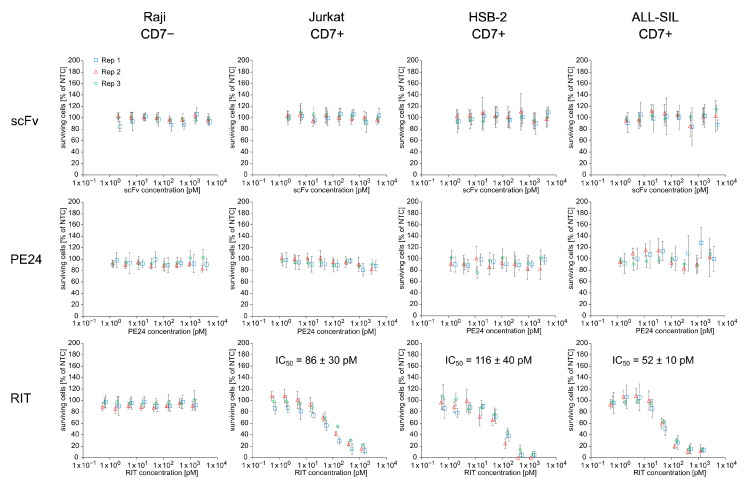
Cytotoxicity of *E. coli* cell-free-produced proteins with GroEL/GroES chaperones. MTT assay of scFv, PE24, and RIT on CD7-negative Raji cells and CD7-positive Jurkat, HSB-2, and ALL-SIL cells. Absorbance values of samples were normalized in respect to absorbance of no-template control (NTC), which was identically diluted and treated as the sample. The data were derived from three independent experiments (rep 1, rep 2, rep 3), which are depicted in different colors and symbols with error bars representing the standard deviation of normalized cell survival performed in triplicate. IC_50_ value for RIT on CD7-positive cell lines was calculated by a four-parameter non-linear regression model. Abbreviations: scFv: single-chain variable fragment; PE24: 24 kDa variant of Pseudomonas Exotoxin A; RIT: recombinant immunotoxin; Rep: replicate.

To evaluate the cytotoxic potential of the *E. coli* cell-free-produced RIT, an MTT assay was performed using unpurified SN fraction in an 8-point dilution series on CD7-negative Raji and CD7-positive Jurkat, HSB-2, and ALL-SIL cells (Figure 3). The RIT showed a concentration-dependent killing of CD7-positive cells with an IC_50_ value of 86 ± 30 pM for Jurkat, 116 ± 40 pM for HSB-2, and 52 ± 10 pM for ALL-SIL cells. In contrast to the effect of the RIT, CD7+ cell lines were not affected by the targeting domain (scFv) or the toxin domain (PE24) themselves. No cytotoxic effects of RIT, scFv, and PE24 were detected for CD7-negative Raji cells. MTT assay-related controls and NTC absorbance values are presented in the Supplementary Material (Figure S5A,B).

### 2.2. CHO-Based Cell-Free Production of PE24 RIT

**Figure 4:**
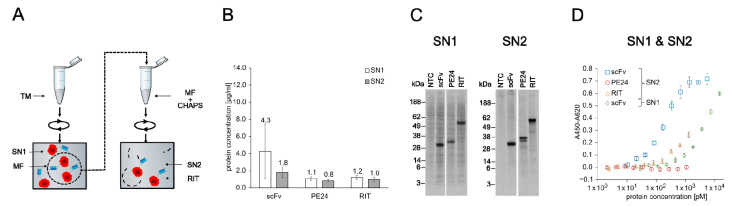
Quantification, autoradiography, and antigen binding of CHO-based cell-free-produced proteins. (**A**) Schematics of CHO cell-free protein synthesis. The RIT was produced in reaction vessels containing the translation mix (TM). TM contained ER-derived microsomes that can be targeted by addition of an N-terminal signal peptide from the honey bee toxin melittin. After synthesis, the TM was centrifuged and the supernatant (SN1) was removed. The remaining pellet represents the microsomal fraction (MF) and was resuspended in the mild detergent CHAPS, which disrupted the microsomes and released the translocated proteins. The resuspended MF was then spun down and the corresponding supernatant (SN2) was removed. SN1 and SN2 were used for downstream assays. (**B**) Liquid scintillation counting for quantification of scFv, PE24, and RIT in SN1 and SN2 fraction. Error bars represent the standard deviation of four independent experiments each performed in triplicate. (**C**) Autoradiograph of reducing denatured 10% Bis-Tris SDS-PAGE. Expected molecular weights are 31.8 kDa for scFv, 31.0 kDa for PE24, and 58.9 kDa for RIT. (**D**) Indirect ELISA of scFv, PE24, and RIT against CD7. Absorbance values in wells without antigen were subtracted from absorbance values in wells coated with CD7. Error bars represent the standard deviation of triplicate analysis. Abbreviations: TM: translation mix; SN: supernatant; MF: microsomal fraction; CHAPS: 3-((3-cholamidopropyl) dimethylammonio)-1-propanesulfonate; scFv: single-chain variable fragment; PE24: 24 kDa variant of Pseudomonas Exotoxin A; RIT: recombinant immunotoxin.

PE inhibits protein synthesis in eukaryotic cells by ADP-ribosylation of the diphthamide residue at position 715 of eEF2 [53,54]. Therefore, eukaryotic expression systems are considered to be unsuitable for PE-based RIT production [10]. However, the CHO cell-free system used here contains ER-derived microsomes in which microsome-translocated proteins are physically separated from the translation apparatus by the microsomal lipid bilayer. We speculated that this might shield eEF2 from PE-mediated ribosylation, thereby allowing the synthesis of sufficient protein amounts for in vitro testing of the RIT produced in a eukaryotic cell-free system. To assess this assumption, we synthesized the anti-CD7 scFv, the toxin domain PE24, and the scFv-PE24 RIT in the microsome-containing CHO cell-free system for quantification of translocated proteins. Afterwards, the completion of CFPS, SN1, and SN2 fractions was prepared (Figure 4A). SN1 represents the soluble fraction of non-translocated proteins and SN2 represents the soluble fraction of translocated proteins. Soluble yields determined by liquid scintillation counting were in a similar range in SN1 and SN2, with 0.8 to 1.2 µg/mL for PE24 and RIT and 4.3 µg/mL (SN1) and 1.8 µg/mL (SN2) for the scFv alone (Figure 4B). SDS-PAGE followed by autoradiography showed distinct signals at the expected molecular weight for scFv and RIT. In SN1, a higher background was detected than in SN2, including the NTC. The higher background likely originates from residual endogenous mRNA present in the lysate. For PE24 a second band with a similar size was detected in SN2 fraction, which indicates N-glycosylation of PE24 since an according glycosylation motif is present in its sequence (Figure 4C).

An essential prerequisite for the efficacy of cytosolic active RITs is their cell entry [27]. Therefore, the antibody domain of the anti-CD7 PE24 RIT is required to bind its target antigen CD7. CD7 binding of the RIT, the scFv alone, and PE24 alone in SN2 was analyzed by ELISA (Figure 4D), including an NTC (Figure S6). To evaluate whether SN1 (not translocated to microsomes) or SN2 (translocated to microsomes) resulted in scFv with higher activity, we additionally performed an ELISA with scFv and NTC from SN1. Next, 10-point serial dilutions of unpurified SN1 and SN2 fractions were prepared. The results show that the scFv from both fractions bound its antigen as indicated by the binding curve. However, while the SN2 scFv binding curve reached saturation at high antibody concentrations with an IC_50_ value of 0.20 nM, the SN1 scFv did not reach saturation with a calculated IC_50_ value of 5.34 nM. This indicated a reduced binding activity of SN1 scFv compared to SN2 scFv. The RIT demonstrated binding to CD7 but saturation was not reached with an IC_50_ value of 0.57 nM. For NTC and PE24 no binding was detected.

**Figure 5:**
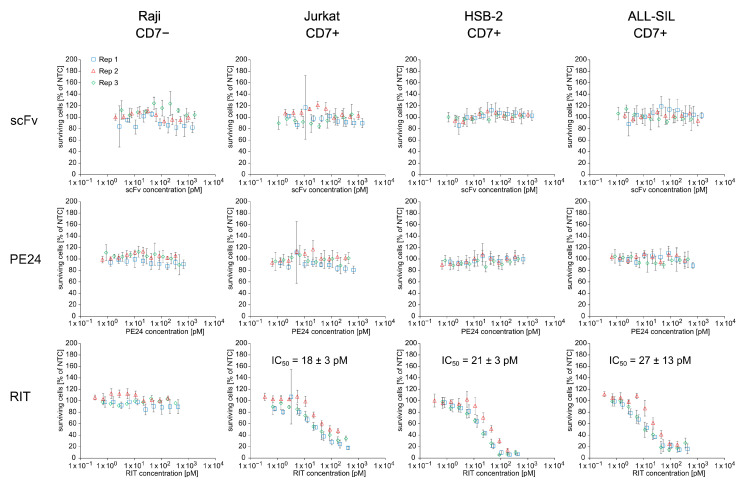
Cytotoxicity of CHO cell-free-produced proteins. MTT assay of proteins from SN2 on CD7-negative Raji cells and CD7-positive Jurkat, HSB-2, and ALL-SIL cells. Absorbance values of samples were normalized in respect to absorbance of no-template control (NTC), which was identically diluted and treated as the sample. The data were derived from three independent experiments (rep 1, rep 2, rep 3), which are depicted in different colors and symbols with error bars representing the standard deviation of normalized cell survival performed in triplicate. IC_50_ values for RIT on CD7-positive cell lines were calculated by a four-parameter non-linear regression model. Abbreviations: scFv: single-chain variable fragment; PE24: 24 kDa variant of Pseudomonas Exotoxin A; RIT: recombinant immunotoxin; SN: supernatant; Rep: replicate.

To assess whether the cell-free-produced RIT was able to specifically kill cells carrying its target receptor CD7, we performed an MTT assay with NTC, scFv, PE24, and RIT on Raji (CD7−), Jurkat, HSB-2, and ALL-SIL (CD7+) cell lines (Figure 5 and Figure S7). For each sample, a 10-point dilution series was directly prepared from unpurified SN2. The results show that the scFv-PE24 RIT caused concentration-dependent cell killing on the CD7-positive cell lines Jurkat, HSB-2, and ALL-SIL but not on CD7-negative Raji cells. ScFv and PE24 did not show a killing effect on any of the cell lines, independent of their CD7 phenotype. The IC_50_ values of the RIT determined on CD7-positive cell lines were 18 ± 3 pM for Jurkat, 21 ± 3 pM for HSB-2, and 27 ± 13 pM for ALL-SIL cells.

To summarize, the anti-CD7 scFv-PE24 RIT, the corresponding scFv, and the corresponding toxin domain PE24 were successfully synthesized in the *E. coli* cell-free system supplemented with ELS as well as in the CHO cell-free system containing microsomes. The *E. coli* cell-free system supplemented with the chaperone system ELS turned out to be the most productive with regard to yield of soluble protein for scFv and RIT. For CHO CFPS, scFv translocated to ER-derived microsomes showed higher binding activity than non-translocated scFv. As shown by ELISA, the RIT synthesized in both cell-free systems specifically bound CD7 while PE24 and NTC did not. In an MTT assay, *E. coli* and CHO cell-free-produced RIT effectively killed CD7-positive cells, while the CD7-negative cell line was unaffected. Furthermore, the scFv targeting domain and the PE24 toxin domain alone did not kill cells, independent of their CD7 phenotype. The data demonstrate that the model RIT could be produced in an active form in the *E. coli* and the CHO cell-free system within a few hours. The purification-free workflow revealed specific binding of the RIT to its target antigen CD7 as well as the specific and effective killing of target-positive cells. *E. coli* CFPS produced a 45 times higher soluble yield of RIT. CHO CFPS produced a 4× higher cytotoxic activity of RIT. Both systems proved suitable for the cell-free-produced RIT in vitro characterization to evaluate antigen binding and cytotoxicity.

## 3. Discussion

RITs are powerful anti-cancer agents as three FDA approvals demonstrate [7]. The FDA-approved RITs are produced in *E. coli*, purified, and refolded from IBs [14,15,16]. During the development process of RITs, repeated sequence optimization of the targeting domain and the linker and toxin domain is required [55]. Therefore, IB solubilization and purification represent a serious bottleneck for RIT development as it is time-consuming and laborious [11]. The open nature, short reaction times, and the option to use linear DNA templates instead of plasmids make CFPS quick and flexible for scalable protein production. To streamline RIT research and development, we explored CFPS for on-demand RIT synthesis and functional characterization with regard to antigen binding and cytotoxicity. To show the proof of concept, we used an anti-CD7 RIT based on PE24 produced in cell-free systems based on CHO and *E. coli* BL21 Star^TM^ (DE3) with ELS. Both cell-free systems allowed the production of active RITs for purification-free in vitro testing in ELISA (Figure 2 and Figure 4) and MTT assay (Figure 3 and Figure 5).

### 3.1. Chaperone Screening for Soluble Yield and Activity of PE24 RIT in E. coli Cell-Free System

*E. coli* cell-free systems are considered the most productive and most widespread cell-free systems used for non-glycosylated therapeutic proteins [42]. To evaluate cell-free RIT synthesis, we therefore chose the commonly applied *E. coli* expression strain BL21 Star^TM^ (DE3) as the parental cell line for the *E. coli* cell-free system. The strain provides enhanced RNA stability and encodes an inducible T7 polymerase, thereby circumventing the need for a separate addition of T7 polymerase when performing the cell-free reaction [56]. *E. coli* cell-based expression and *E. coli* cell-free expression often result in low yields of soluble protein, particularly for complex proteins of eukaryotic origin but also for many proteins of bacterial origin [57,58,59]. The scFv domain of the RIT is of murine origin [60], which might in part explain the low yields of scFv and RIT in the *E. coli* cell-free system based on wildtype BL21 Star^TM^ (DE3). However, as mentioned above, insoluble protein formation is a general issue in *E. coli* cell-based and cell-free overexpression [48,61].

For *E. coli* cell-based heterologous protein expression, soluble yields and protein activity can be increased by the co-expression of chaperones [47]. The same holds true for *E. coli* CFPS. In the past, chaperones were either added directly to *E. coli* S30-based cell-free systems [62,63,64,65,66,67,68,69,70,71,72,73,74] or to *E. coli* reconstituted cell-free systems [75,76,77,78,79]. Alternatively, chaperones can be supplemented by overexpression in the *E. coli* host used for the S30 cell-free system preparation [80,81,82,83,84]. TF, KJE, and ELS are among the major, best-characterized chaperone systems in *E. coli*. They have distinct modes of action and substrate preference but act synergistically with overlapping function. They provide means to prevent and resolve unproductive folding by scanning of the folding landscape to find the correctly folded native state. However, as of yet, the exact mechanism of folding network cooperativity of TF, KJE, and ELS and their substrate characteristics are not well-understood [45,85]. Therefore, identification of a suitable chaperone for a given protein currently remains a trial and error process [76,86].

Here, commercially available plasmids, coding for the chaperones KJE+ELS, ELS, KJE or TF+ELS, were transformed into the BL21 Star^TM^ (DE3) strain to avoid separate expression and purification of the individual chaperones. Based on the transformed strains, lysates were produced, which accordingly contained T7 polymerase and the respective chaperones. CFPS of scFv and RIT showed that the chaperones significantly increased the yield of soluble scFv and scFv-PE24 RIT for *E. coli* CFPS system. Thus, chaperone levels already present in the BL21 Star^TM^ (DE3) wildtype strain were not sufficient to provide a productive folding environment for the cell-free-produced proteins. The highest yields of soluble scFv and RIT were obtained when providing additional amounts of chaperones ELS and KJE alone or in combination. These results are in agreement with a study by Niwa et al. who analyzed 800 aggregation-prone cytosolic *E. coli* proteins in a reconstituted *E. coli* cell-free system and found significant overlap for solubility increase by ELS and KJE [76]. Based on the results of this study, we decided to not evaluate TF alone in our study although available in the chaperone plasmid set as only 5 of the 800 analyzed proteins in the paper by Niwa et al. benefitted exclusively from TF-only addition [76]. In *E. coli*, TF acts in a 1:1 molar ratio with ribosomes on substrates but is present in a 1–2× molar excess over ribosomes. Therefore, one study argued to disregard TF when screening for chaperones since wildtype TF levels would be sufficient without substantiating their claim by experimental data [87]. Another study proposed to exclude TF from screening for scFv solubility since TF addition did not result in higher solubility [88]. In contrast, for an artificial α-helix-rich protein, TF supplementation alone resulted in a solubility increase similar to ELS supplementation [89]. Furthermore, Niwa et al. found that some of the studied proteins required the concerted action of TF, KJE, and ELS for increased solubility. However, said study was conducted in a reconstituted cell-free system without any basal level of TF present [76]. In our study, TF+ELS addition yielded lower soluble protein for cell-free-produced scFv and RIT compared to ELS addition alone. This indicates that the soluble yields of the proteins examined here do not benefit from additional amounts of TF, at least when elevated levels of ELS are provided. In addition to increasing soluble yield, some studies also reported on increased activity of the POI by supplementation of ELS, KJE, or TF in *E. coli* cell-free systems [62,63,66,80] and for *E. coli* cell-based expression [47]. In contrast, another study reported that the increase in soluble protein was due to the solubilization of inactive inclusion bodies in *E. coli* cells without a gain in activity [90]. In our study, both scFv and RIT activity were increased by ELS addition compared to the cell-free system without additional amounts of chaperones. In contrast, KJE+ELS slightly decreased the activity of scFv but showed similar activity for the RIT. Although TF+ELS as well as KJE increased soluble yield, the activity of the scFv and RIT was reduced compared to the cell-free system with wildtype chaperone levels. The results indicate that ELS is the most beneficial chaperone for the cell-free-produced scFv and RIT examined here. However, the literature suggest that the gain in soluble yield and activity from different chaperones is POI-specific. Thus, it is advisable to screen for the optimal chaperones for a given POI in order to reach the highest possible yield of soluble and functional protein. Further research is required to rationalize the characteristics of a given protein that determine which chaperone is most efficient.

Based on the chaperone screening, the *E. coli* cell-free system with ELS was chosen for assessing antigen binding and cytotoxicity of the RIT. The evaluation of RIT, scFv, and PE24 antigen binding indicate that the scFv portion of the RIT mediates CD7 binding. A cytotoxicity assay with RIT, scFv, and PE24 showed that the RIT is active and specifically kills cells expressing its target receptor in a concentration-dependent manner. We conclude that the *E. coli*-based cell-free system with ELS is suitable for production and in vitro testing of the model RIT. To our knowledge, this study reports RIT production in an *E. coli* cell-free system for the first time.

### 3.2. Production of PE24 RIT in CHO Cell-Free System

The place of action of Pseudomonas Exotoxin A and its derivatives is the cytosol of eukaryotic cells where it ribosylates the diphthamide residue 715 of eEF2, thereby leading to translation inhibition and subsequent cell death [22]. PE is known to be a highly effective translation inhibitor. It has been postulated that one molecule of PE in the cytosol is sufficient to kill a cell [91]. Despite the susceptibility of eukaryotic translation towards PE-mediated inhibition, the CHO cell-free system used in this study yielded sufficient amounts of scFv-PE24 RIT for its in vitro characterization. We propose that the microsomes contained in the CHO cell-free system provide a “shielding effect” since microsome-translocated proteins are physically separated from the translation machinery. This likely contributed to the adequate yield that was observed in this study for RIT synthesis in the CHO cell-free system. However, a substantially high fraction of RIT was not translocated, eventually halting protein synthesis by self-inhibition. This resulted in a limited yield of RIT and PE24 in both SN1 and SN2 fractions compared to scFv. We expect that a higher fraction of translocated RIT molecules to the microsomes would further impede PE-mediated eEF2 ribosylation, which in turn would lead to higher yields in SN2. Co-translational ER translocation is mainly achieved by the Sec61 translocon system. Therefore, Sec61 and its associated proteins are attractive targets to achieve more efficient microsome translocation in CHO cell-free systems. Cell line engineering of the parental CHO strain could be employed to overexpress key players involved in translocation, such as Sec61 itself, signal recognition particle, TRAP complex, or Hsp70/40 members DnaK/J [92]. Furthermore, to increase the yield of PE-derived proteins in the CHO cell-free system, addition of exogenous eEF2 to the cell-free reaction would likely outcompete its PE-mediated inhibition. Alternatively, the addition of PE-inhibiting molecules might increase protein yield. Several PE inhibitors have been identified in the past [93,94,95,96,97,98,99]. However, these inhibitors would need to be removed after synthesis to obtain active RIT. Furthermore, cell line engineering of eEF2 in the parental CHO cell line might be expedient to render the cell-free system less susceptible to PE24 action. Possible targets include the genes involved in diphthamide biosynthesis in eEF2, such as DPH1, DPH2, DPH5, and DPH7 [100,101,102] or eEF2 itself [103,104].

To our knowledge, the only published application of CFPS for RIT production was established in the early 1990s in a rabbit reticulocyte lysate (RRL) using an anti-transferrin receptor scFv linked to PE and DT [105]. The study did not report yields for cell-free-produced RITs and therefore cannot be used to reference soluble yields determined in this paper. The IC_50_ value for protein synthesis inhibition in K562 cells was reported to be in the low nM range, which is comparable to the IC_50_ value determined here for cytotoxicity by MTT assay. However, from an ethical point of view, the manufacturing process for RLL-based cell-free systems is questionable since it involves the euthanization of animals [106]. In contrast, CHO cell-free systems are derived from in vitro cultured cells [107]. Therefore, at a similar performance and with ethical reasons considered, eukaryotic cell-free RIT production in CHO-based cell-free systems should be preferred over RRL-based cell-free systems. Here, we report RIT production in a CHO cell-free system for the first time.

A high-affinity targeting domain has been shown to be beneficial for RIT efficacy on leukemic tumors and in vitro cell culture [20,108,109,110]. In contrast, solid tumors require a careful balancing of RIT affinity due to an effect called the binding-site barrier [17,111]. In this study, a high-affinity RIT was desirable since CD7 is associated with target cells from the hematopoietic lineage. An anti-CD7 ELISA was performed to evaluate whether CHO cell-free synthesized scFv obtained from fraction SN1 (not microsome-translocated) or the scFv obtained from fraction SN2 (microsome-translocated) was more active. SN1 scFv did not reach saturation in ELISA, with an IC_50_ value of 5.34 nM, while the IC_50_ value of SN2 scFv was determined to be 0.20 nM. This indicates that SN2 scFv is more active than SN1 scFv. Similar observations for CHO CFPS have been made with an anti-SMAD scFv-Fc and an anti-EGFR scFv [50,51]. This effect can be explained by the folding promoting environment in the microsomes from which SN2 antibodies are derived. We confirmed that SN2 fraction is superior over SN1 fraction for RIT activity by an MTT assay with CD7-positive Jurkat and HSB-2 cells. In that case, SN2 RIT killed cells more effectively than SN1 RIT. Accordingly, proteins from SN2 fraction were used for CHO-based cell-free RIT production and testing. We showed that antigen binding is mediated by the scFv domain of the RIT and that the CHO cell-free-produced RIT is active and specifically kills cells expressing the target receptor. Despite the susceptibility of CHO-based translation to the toxic mechanism of PE, sufficient amounts of the PE-based RIT could be produced in the CHO cell-free system for RIT in vitro testing. We conclude that the CHO cell-free system containing microsomes is suitable for RIT screening purposes.

### 3.3. Comparison of CHO and E. coli Cell-Free-Produced PE24 RIT

Concurrent results were obtained for the scFv, PE24, and RIT synthesized in the *E. coli* cell-free system with ELS and the CHO cell-free system with respect to analysis by ELISA and MTT assay. Both the *E. coli* and CHO cell-free synthesized scFv and the RIT bound CD7 in the indirect ELISA while PE24 and NTC did not. The IC_50_ value of the scFv produced in the *E. coli* cell-free system with ELS was around 1 nM and thereby approximately five-fold higher than the CHO cell-free-produced scFv from SN2. This signifies lower activity of the scFv produced in the *E. coli* cell-free system compared to the CHO cell-free system. Congruently, the IC_50_ value determined in ELISA for the RIT synthesized in the *E. coli* cell-free system with ELS was around four-fold higher than the RIT derived from CHO SN2. Cell-free-produced RIT from the *E. coli* and the CHO cell-free system effectively killed CD7+ Jurkat, HSB-2, and ALL-SIL cells. The CD7-negative control cell line was not affected. Furthermore, none of the cell lines, including the CD7− Raji control cell line, were affected by the individual scFv domain and the PE24 toxin domain. Therefore, it can be concluded that the RIT specifically killed cells by binding the CD7 receptor via its scFv domain. It is plausible that CD7-bound RIT has been internalized and the toxin domain was trafficked from late endosome or early lysosome in a retrograde manner via Golgi and ER into the cytosol. In that case, RIT ribosylated eEF2, thereby halting protein synthesis and leading to cell death [27]. The IC_50_ values determined in the MTT assay was 18 ± 3 pM, 21 ± 3 pM, and 27 ± 13 pM for CHO CFPS-produced RIT and 86 ± 30 pM, 116 ± 40 pM, and 52 ± 10 pM for *E. coli* CFPS-produced RIT on CD7-positive Jurkat, HSB-2, and ALL-SIL cells, respectively. Thus, the IC_50_ values of *E. coli* CFPS were roughly four-fold higher than IC_50_ values from CHO CFPS, similar to the observation made for the scFv domain. For the given RIT and scFv, it can be concluded that CHO cell-free-produced proteins were more active than *E. coli* cell-free-produced proteins. The folding environment in the ER-derived microsomes of the CHO system is likely more beneficial for RIT activity than the *E. coli* cell-free reaction even when supplemented with ELS. However, the *E. coli*-based cell-free system with ELS yielded 45 times higher soluble yields compared to the CHO-produced RIT from SN2. Therefore, less metabolic burden is imposed on the chaperone system in the CHO cell-free system. However, as elucidated by the autoradiographs presented here, *E. coli* CFPS is known to yield cessation products, particularly for large proteins [59]. These termination products likely originate from the depletion of certain amino acids, particularly serine and cysteine, as well as energy resources, such as CTP, UTP, and ATP [112,113]. Therefore, liquid scintillation quantification overestimates the actual amount of full-length target protein. Based on a densitometric analysis of the autoradiograph background against the signal of the POI, the IC_50_ value of the *E. coli* cell-free synthesized RIT might be up to 2.3-fold lower. This demonstrates the need for the cautious interpretation of data when working with unpurified cell-free synthesized protein mixtures. In contrast, CHO CFPS yields distinctly fewer side products, particularly in SN2 fraction. Moreover, due to cheaper materials, faster growth rates, and a simpler manufacturing process, production of the *E. coli* cell-free system is considerably more cost-effective than the CHO cell-free system. On the other hand, proteins are endotoxin-free when derived from the CHO cell-free system. Yields in the CHO cell-free system might be increased further by the above-mentioned strategies to strengthen its PE tolerance.

### 3.4. Anti-CD7-Targeted T Cell Depletion

The pan T cell marker CD7 was chosen as the target for the antibody–toxin conjugate used here, since CD7+ T cells and B cells are associated with a multitude of diseases, such as most cases of T-ALL [29], some cases of AML [30], and B-ALL [31,114], as well as GvHD [32,33]. AML and B-ALL have recently become manageable in a clinical setting [115,116] but treatment options for relapsed and refractory T-ALL and GvHD remain limited despite promising drugs in clinical trials [117,118]. Further treatment options are required for all mentioned diseases. An RIT targeting CD7 might particularly be useful for the treatment of relapsed and refractory T-ALL or GvHD, aiming to bridge patients to HSCT. Anti-CD7 drugs that are currently being evaluated in clinical trials are CAR-T and CAR-NK cell drugs for T-ALL [119,120,121,122] and a combination of Ricin-conjugated anti-CD3/CD7 immunotoxins for GvHD [123]. In the past, an anti-CD7 immunotoxin linked by disulfide bonds to Ricin was not effective in a phase I clinical trial with patients with CD7+ T cell leukemia and lymphoma [124], most probably due to dose-limiting toxicities. Several other anti-CD7 antibody–toxin conjugates did not or have not yet entered clinical trials [125,126,127,128,129,130,131,132,133,134,135,136,137]. CD7 is present on healthy peripheral T cells and NK cells and their precursors [28]. However, a subset of CD8+ T cells is CD7-negative [33], thereby potentially leaving immunocompetent T cells after CD7-targeted cell depletion [119]. Still, CD7 targeting leads to the depletion of the majority of T cells, which brings considerable risk for potentially life-threatening opportunistic infections and would need to be managed according to established HSCT protocols [9,138].

Considering the widespread use of PE-derived toxin domains in RIT development [27] and challenges for the expression of PE-derived RITs in eukaryotic expression hosts, the anti-CD7 RIT based on PE24 used in this paper represents an insightful model protein. We show that CFPS allows quick, flexible, on-demand synthesis of an RIT and demonstrate the proof-of-concept for RIT in vitro testing using a CHO-based and an *E. coli*-based cell-free system. We anticipate that cell-free protein synthesis may be used as a platform technology to accelerate RIT research and development.

## 4. Materials and Methods

### 4.1. DNA Templates for Cell-Free Protein Synthesis

#### 4.1.1. Regulatory Elements

Plasmids with pUC57-1.8k backbone were synthesized by Biocat GmbH (Heidelberg, Germany) and used either directly as a template for CFPS or as a template for polymerase chain reaction (PCR, see below). The 5′ untranslated region (UTR) of templates used for *E. coli* CFPS contained a T7 promotor for mRNA generation and a Shine-Dalgarno sequence for prokaryotic translation initiation followed by a *NcoI* restriction site with ATG start codon in-frame and a 3′ UTR with a T7 terminator. Plasmids used for CHO CFPS contained a T7 promotor in the 5′ UTR and an internal ribosomal entry site (IRES from the intergenic region of the cricket paralysis virus) to allow for cap-independent translation initiation. The 3′ UTR contained a T7 terminator. Within this study, all genes of interest for CHO CFPS were N-terminally fused to the signal sequence of honey bee toxin melittin. The melittin signal peptide allows for translocation of the respective proteins into the lumen of the ER-derived microsomes present in the cell-free translation mix. The coding sequences of the genes of interest were codon-optimized for *Cricetulus griseus* codon usage (excluding the melittin signal sequence) or for *E. coli* codon usage using GeneOptimizer algorithm [139]. DNA sequences are listed in Figures S8 and S9.

#### 4.1.2. ScFv-Targeting Domain

The scFv in V_L_-V_H_ orientation is of murine origin, targets CD7, and is based on the sequences included in patent WO2003/051926. Downstream of V_H_, a sequence coding for a C-terminal Twin Strep Tag for ELISA detection was added.

#### 4.1.3. PE24 Toxin Domain

The sequence of PE24 was constructed starting from the full-length sequence of Pseudomonas Exotoxin A (PE66), which targets LRP-1 (CD91) receptor. The PE66 sequence was retrieved from NCBI RefSeq NP_249839.1. The native signal peptide (amino acid 1 to 25) was removed, the C-terminal ER localization signal REDL (amino acids 609 to 612) was replaced by KDEL for higher activity, and the most C-terminal amino acid K613, which impairs KDEL receptor binding, was removed [140]. For construction of the de-immunized PE24 variant without native binding domain [26], the sequence coding for amino acids 1 to 273 and 285 to 394 of domain II were deleted, leaving the furin cleavage site intact. A sequence coding for a Twin Strep Tag was inserted at the 5′ end (i.e., downstream of the melittin signal peptide for CHO CFPS or downstream of the *NcoI* site for *E. coli* CFPS). The amino acid numbering used here is relative to the mature PE66 protein without signal peptide.

#### 4.1.4. Recombinant Immunotoxin

For construction of the RIT (scFv-PE24), the sequences of the scFv with C-terminal Twin Strep Tag and PE24 without N-terminal Twin Strep Tag were connected by 60 bp coding for a GS-rich linker. Therefore, the RIT carries its Twin Strep Tag between the scFv and the PE24 domain.

### 4.2. DNA Template Generation by Plasmid Prep and PCR

Generation of *E. coli* strains carrying plasmids that contain a toxin domain is cumbersome due to regulatory safety restrictions. Therefore, templates for cell-free synthesis of PE24 and RIT were generated by PCR, while a plasmid was used for cell-free synthesis of the scFv. The plasmid coding for the scFv was prepped from an overnight liquid culture of a single clone after transformation of electrocompetent *E. coli* JM109 using PureLink™ HiPure Plasmid Midiprep Kit (Thermo Fisher Scientific, Waltham, MA, USA), according to the manufacturer’s instructions.

PCRs were carried out in 60 µL aliquots using 0.04 ng/µL of the respective plasmid with Q5^®^ Hot Start High-Fidelity DNA Polymerase and dNTP Solution Mix (NEB, Ipswich, MA, USA), with primer A (5′-ATGATATACGTACGATAGGCTAGC) and primer B (5′-ACCCCTCAAGACCCG), and at an annealing temperature of 60 °C and for 35 cycles, according to manufacturer’s recommendations, in a Biometra TRIO thermocycler (Analytik Jena, Jena, Germany). A total of 1 µL of the PCR reaction was analyzed by electrophoresis in a 1.2% TBE-agarose gel and then 500 µL of the PCR reaction was purified using DNA Clean and Concentrator-25 (Zymo Research, Irvine, CA, USA), with elution in 50 µL of ultra-pure water, and concentration was measured using a Nanodop-2000c spectrophotometer (Thermo Fisher Scientific, Waltham, MA, USA).

### 4.3. Cell-Free Protein Synthesis

#### 4.3.1. *E. coli*-Based Cell-Free Protein Synthesis

Cell extract was prepared from *E. coli* BL21 Star^TM^ (DE3) (Invitrogen, Carlsbad, CA, USA) and was transformed by heat shock with the plasmids pG-KJE8, pGro7, pKJE7, or pG-Tf2 (Takara Bio, Mountain View, CA, USA) for chaperone expression. Plasmids carry the genes for the bacterial chaperones trigger factor (TF), DnaK/DnaJ/GrpE (KJE) and GroEL/GroES (ELS) in different combinations under control of araB or Pzt-1 promoter. Strain BL21 Star™ (DE3) contains the λDE3 lysogen for T7 polymerase expression under control of the lacUV5 promotor induced by isopropyl β-D-1-thiogalactopyranoside (IPTG).

Cells were grown in 2 L shaking flasks at 37 °C and 200 rpm in 2×YPTG (pH 7) medium containing 10 g/L yeast extract, 16 g/L tryptone, 5 g/L NaCl, and 20 g/L glucose. Cell growth was monitored online with optical Sensors (Cell Growth Quantifier CGQ, Aquila Biolabs, Baesweiler, Germany) to induce overexpression and harvest the cells at defined growth phases. Overexpression of chaperones was induced with 4 mg/mL L-arabinose (araB promotor) and/or 10 ng/mL tetracycline (Pzt-1 promotor) according to the manufacturer’s manual at the beginning of the log phase. One hour later, overexpression of T7 RNA polymerase was induced with 1 mM IPTG. Cells were harvested in the late log phase after a total cultivation time of 4 h [141]. After cultivation, the culture was poured into a 0.4 L centrifuge bottle, cooled down on ice for 20 min, and centrifuged at 3000× *g* for 10 min at 4 °C. The cell pellet was washed twice with buffer A (0.1 M HEPES, pH 7.6; 0.5 M potassium acetate; 0.5 M ammonium acetate; 0.1 M MgCl_2_; 50 M EDTA; 1% NaN_3_), shock-frozen in liquid nitrogen, and stored at −80 °C. For extract preparation, cells were thawed on ice and resuspended with 1 mL buffer A (+2 mM DTT) per 1 g of cell pellet. Cells were disrupted with a high-pressure homogenizer Cell Disrupter CF1 (Constant Systems, Daventry, UK) at 25,000 psi at 2 °C [142]. Disrupted cells were centrifuged at 18,000× *g* for 10 min at 4 °C. The supernatant was purified from endogenous amino acids and concentrated with the cross-flow filtration device Äktaflux with 10 kDa cross-flow filter UFP-10-C-2U (Cytiva, Marlborough, MA, USA) at 2 °C. The lysate was shock-frozen in liquid nitrogen and stored at −80 °C.

Cell-free reactions contained (f.c.) 26% of purified lysate, 51.8 mM HEPES, 3.25 mM DTT, 10 µg/mL aproptinin, 5 µg/mL leupeptin, 5 µg/mL pepstatin (Roche, Basel, Switzerland), 0.1 U/µL RNase inhibitor (Promega, Fitchburg, MA, USA), 100 μg/mL bulk yeast tRNA, 8 µg/mL pruvate kinase (Roche, Basel, Switzerland), 11 mM MgCl_2_, 2.5 mM PEG3000, 0.01% NaN_3_, 0.06 mM folinic acid (Sigma-Aldrich, St. Louis, MO, USA), 0.75 mM of each canonical amino acids except leucine and tyrosine (Merck, Darmstadt, Germany), 0.75 mM tyrosine in KOH, 0.75 mM leucine, 1.1 mM ATP, 1.1 mM GTP, 0.55 mM CTP, 0.55 mM UTP, 31.25 mM phosphoenolpyruvate (Roche, Basel, Switzerland), 73.7 mM potassium acetate, 36.8 mM ammonium acetate, 10.6 mM acetylphosphate, and 74 µM EDTA (Sigma-Aldrich, St. Louis, MO, USA).

For subsequent qualitative and quantitative analysis by autoradiography and liquid scintillation counting, ^14^C-leucine (f.c. 50 μM) was added to cell-free reactions (specific radioactivity 12.6 dpm/pmol, Perkin Elmer, Waltham, MA, USA). To start the reaction, plasmid or PCR product (f.c. 20 nM) was added and the mixture was incubated for 2 h at 30 °C with 500 rpm agitation on Thermomix Comfort (Eppendorf, Hamburg, Germany).

After completion of cell-free synthesis, an aliquot of the translation mix (TM) was transferred to a fresh tube for quantitative and qualitative analysis and the reaction was centrifuged at 16,000× *g* for 30 min at 4 °C. The supernatant was transferred to a fresh tube representing the soluble fraction of cell-free synthesized target proteins (SN).

#### 4.3.2. CHO-Based Cell-Free Protein Synthesis

Translationally active CHO lysates were used for CHO-based CFPS as previously described [143]. In brief, three different premixed buffers (A, B, and C) were combined. Premix A (10×) contained 300 mM HEPES-KOH, pH 7.6, 1 mM of each canonical amino acids except leucine and tyrosine (Merck, Darmstadt, Germany), 1 mM tyrosine in KOH, 1 mM leucine, 2.5 mM spermidine, 39 mM Mg(OAc)_2_, and 1350 mM KOAc. Premix B (2.5×) was prepared as previously described [107], containing S7 nuclease-treated CHO lysate supplemented with 250 μg/mL creatine kinase and 50 μg/mL bulk yeast tRNA (Roche, Basel, Switzerland). Premix C (5×) contained 100 mM creatine phosphate, 8.75 mM ATP, 1.5 mM CTP, 1.5 mM UTP, 1.5 mM GTP (Roche, Basel, Switzerland), and 0.5 mM m^7^G(ppp)G cap analogue (Prof. Edward Darzynkiewicz, Warsaw University, Poland). On ice, premixes A, Bm=, and C were diluted to 1× concentrated solution in ultra-pure water and supplemented with 1 U/μL (f.c.) T7 RNA polymerase (Agilent, Santa Clara, CA, USA). For subsequent qualitative and quantitative analysis by autoradiography and liquid scintillation counting, ^14^C-leucine (f.c. 50 μM) was added to cell-free reactions (specific radioactivity 66.67 dpm/pmol, Perkin Elmer, Waltham, MA, USA). To start the reaction, plasmid or PCR product (f.c. 20 nM) was added and the mixture was incubated for 3 h at 30 °C with 600 rpm agitation on Thermomix Comfort (Eppendorf, Hamburg, Germany).

After completion of CFPS, the reaction was centrifuged at 16,000× *g* for 30 min at 4 °C. The supernatant was transferred to a fresh tube representing the soluble fraction of cell-free synthesized non-translocated target proteins (SN1). After removal of SN1, the microsomal pellet was washed with 50 µL of phosphate-buffered saline (PBS, pH 7.4) and centrifuged and then the supernatant was discarded. The washed pellet was resuspended in PBS supplemented with 0.5% CHAPS (3-((3-cholamidopropyl) dimethylammonio)-1-propanesulfonate, SERVA Electrophoresis, Heidelberg, Germany) and rigorously agitated for 15 min at RT to release the translocated proteins from the lumen of the microsomal vesicles. To obtain the soluble fraction of microsome-translocated antibodies, the solutions were centrifuged and the resulting supernatant (SN2) was transferred to a fresh tube.

### 4.4. Determination of Total Protein Yields

Liquid scintillation counting was used to quantify cell-free synthesized ^14^C-labeled proteins [106]. Following CFPS and fractionation into TM and SN (*E. coli* CFPS) or SN1 and SN2 (CHO CFPS), triplicates of aliquots of 2 µL (*E. coli* CFPS) or 7 μL (CHO CFPS) were mixed with 3 mL trichloroacetic acid (TCA) and incubated in a 80 °C water bath for 15 min, followed by incubation on ice for 30 min. In order to remove non-incorporated ^14^C-leucine, protein solutions were filtered using a vacuum filtration system (Hoefer, Holliston, MA, USA). Incorporated ^14^C-leucine in cell-free synthesized proteins was measured by liquid scintillation counting using the HIDEX 600 SL (Hidex, Turku, Finland).

### 4.5. SDS-PAGE, Autoradiography

Sodium dodecyl sulfate polyacrylamide gel electrophoresis (SDS-PAGE) [144] was performed using precast 10% Bis-Tris gels (Invitrogen, Carlsbad, CA, USA) or self-made 12% Tris-Glycine gels (SureCast, Thermo Fisher Scientific, Waltham, MA, USA) under reducing, denaturing conditions with subsequent autoradiography to visualize cell-free synthesized ^14^C-labeled proteins [106]. In general, 7 μL aliquots of SN1 or SN2 (CHO CFPS) or 2 µL of TM or SN (*E. coli* CFPS) were filled to 50 µL with ultra-pure water on ice and 150 µL of ice cold acetone was added. For precipitation of proteins, reaction tubes were incubated on ice for 15 min, centrifuged (16,000× *g*, 10 min, 4 °C) and the supernatant was discarded. A total of 15 µL of 1× LDS buffer (Invitrogen, Carlsbad, CA, USA) supplemented with DTT (f.c. 50 mM) was added to the dried precipitated protein pellet, incubated under rigorous shaking at RT for 15 min, followed by incubation at 70 °C for 10 min and loaded onto PAGE gels, which were run for 30 min at 185 V (10% Bis-Tris gels) or 60 min at 150 V (12% Tris-Glycine gels). For autoradiography following electrophoresis, gels were stained with Coomassie Blue (SimplyBlue SafeStain, Thermo Fisher Scientific, Waltham, MA, USA). After staining, gels were dried on Whatman paper for 60 min to 70 min at 70 °C using Unigeldryer 3545D (Uniequip, Planegg, Germany). Radioactively labeled proteins were visualized using the phosphor imager system Amersham Typhoon RGB Biomolecular Imager (GE Healthcare, Chicago, IL, USA).

### 4.6. ELISA

Functional analysis of cell-free synthesized proteins by indirect enzyme-linked immunosorbent assay (ELISA) [145] was performed in triplicate by overnight coating (at 4 °C) a 96-well microtiter plate (Corning, Wiesbaden, Germany) with 20 nM (f.c.) recombinant CD7-His antigen (extracellular domain A26-P180, Sino Biological, Eschborn, Germany) in 50 µL of 0.1 M sodium carbonate buffer pH 9.6 per well. The next day, the plate was washed 3× with 200 µL of PBS-T (PBS containing 0.05% Tween-20). CD7-coated wells as well as empty wells serving as no antigen control (nAg) were blocked with 200 µL of PBS containing 2% BSA (cat# 422381B, VWR, Darmstadt, Germany) for 2 h on a rotary shaker. The washing step was repeated and CD7-coated wells and nAg wells were incubated with 50 µL of cell-free synthesized protein diluted in PBS containing 1% of BSA for 2 h at 300 rpm. For the ELISA binding curves, an 8-point 1:3 serial dilution starting from 1:20 was prepared for *E. coli* cell-free synthesized proteins. For proteins expressed in the CHO cell-free system, a 10-point 1:2 serial dilution starting from a 1:10 dilution was prepared. After another washing step, wells were incubated with 50 µL of Strep-Tactin^®^-HRP (IBA, Göttingen, Germany) diluted to 1:2000 in PBS containing 1% BSA to bind the Twin Strep Tag. After 3× washing with PBS-T, wells were incubated with 100 µL of TMB substrate solution (Thermo Fisher Scientific, Waltham, MA, USA) diluted to 1:2 in ultra-pure water for 8 min. Color development was stopped by adding 100 µL of 0.5 M H_2_SO_4_ and absorbance was measured using a Mithras LB943 plate reader (Berthold, Bad Wildbad, Germany) at 450 nm (reference 620 nm). The A_450–620_ signal of nAg wells was subtracted from the A_450–620_ signal of target-coated wells to obtain a value indicating specific binding against BSA block control. Where applicable, IC_50_ values were calculated by a four-parameter non-linear regression model (Quest Graph™ IC_50_ Calculator, AAT Bioquest, Inc., https://www.aatbio.com/tools/ic50-calculator, accessed on 9 October 2022).

### 4.7. Cell Culture

Raji, Jurkat, HSB-2, and ALL-SIL cell lines were obtained from DSMZ (Heidelberg, Germany) and cultured in medium RPMI1640 with 25 mM Hepes, 2 g/L NaHCO_3_ supplemented with 2 mM stable Gln, 100 µg/mL penicillin/streptomycin (Pan Biotech, Aidenbach, Germany), 1 mM sodium pyruvate (Biowest, Nuaillé, France), and 10% fetal bovine serum (Biochrom, Berlin, Germany). Cells were incubated at 37 °C, 5% CO_2_.

### 4.8. MTT Assay

3-(4,5-dimethylthiazol-2-yl)-2,5-diphenyltetrazolium bromide (MTT) assay [146] was used to assess cell-toxic effects of the cell-free synthesized POIs on Raji (CD7-), Jurkat, HSB-2, and ALL-SIL (CD7+) cell lines. Cells were seeded approximately 20 h before addition of cell-free synthesized proteins in a 96-well plate (Sarstedt, Nümbrecht, Germany) in 90 µL medium containing 5000 cells for Raji and Jurkat and 20,000 cells for HSB-2 and ALL-SIL. Cell-free synthesized proteins from SN fraction (*E. coli* CFPS) and SN2 fraction (CHO CFPS) including a no-template control (NTC) were diluted in PBS containing 0.5% CHAPS. For CHO cell-free expressed proteins, a 10-point 1:2 serial dilution starting from a 1:4 dilution was prepared. For the *E. coli* cell-free synthesized RIT in the chaperone screening, a 10-point 1:3 serial dilution starting from 1:20 was prepared. For the *E. coli* cell-free expression of scFv, PE24, and RIT in presence of ELS, an 8-point 1:3 serial dilution starting from 1:60 was prepared. A total of 10 µL of diluted SN (*E. coli* CFPS) or SN2 (CHO CFPS) fraction was added to the cells and incubated at 37 °C at 5% CO_2_ for 70 h. Then, 10 µL of 5 mg/mL MTT (Merck, Darmstadt, Germany) in PBS (sterile filtered) was added to all wells and incubated for 4 h. To solubilize the formazan crystals, 100 µL of 10% SDS with 10 mM HCl was added and incubated overnight. Spectrophotometric measurement was performed using a Mithras LB943 plate reader (Berthold, Bad Wildbad, Germany) at 570 nm (reference 630 nm). The absorbance was normalized by dividing the absorbance value of the sample by the absorbance of the respective dilution of the NTC sample and multiplied by 100 to obtain the percentage of surviving cells of NTC. The MTT assay was replicated three times. For each biological replicate, the IC_50_ value for RIT on CD7-positive cell lines was calculated using Quest Graph™ IC_50_ Calculator (AAT Bioquest, Inc., https://www.aatbio.com/tools/ic50-calculator, accessed on 9 October 2022). Final IC_50_ value represented the mean of three replicates with its standard deviation. Controls for the MTT assay included addition of medium, the diluent CHAPS (f.c. 0.5%), cell-toxic DDM (n-Dodecyl-β-D-maltoside, f.c. 0.2%), and serially diluted NTC.

## Data Availability

The data presented in this study are available on request from the corresponding author.

## Supplementary Materials

The following supporting information can be downloaded at: https://www.mdpi.com/article/10.3390/ijms232213697/s1.

## Abbreviations

AML: Acute myeloid leukemia; B-ALL: acute B lymphoblastic leukemia; CFE: cell-free expression; CFPS: cell-free protein synthesis; CHO: Chinese hamster ovary; DDM: n-Dodecyl-β-D-maltoside; DT: diphtheria toxin; leukemia; eEF2: eukaryotic elongation factor 2; ELISA: enzyme-linked immunosorbent assay; ELS: GroEL/GroES; ER: endoplasmic reticulum; GvHD: graft vs. host disease; IRES: internal ribosomal entry site; KJE: DnaK/DnaJ/GrpE; NTC: no-template control; PE or PE66: full-length Pseudomonas Exotoxin A; PE24: 24 kDa variant of PE; PE38: 38 kDa variant of PE; POI: protein of interest; RIT: recombinant immunotoxin; SDS: sodium dodecyl sulfate; scFv: single-chain variable fragment; SP: signal peptide; T-ALL: T cell acute lymphoblastic leukemia; TF: trigger factor.
