# Supplementary material for: Synthesis of an Anti-CD7 Recombinant Immunotoxin Based on PE24 in CHO and E. coli Cell-Free Systems
Source: Int J Mol Sci. 2022 Nov 8;23(22):13697. doi: 10.3390/ijms232213697 (PMC9697001; doi:10.3390/ijms232213697)
Supplement: Supplementary file 1 [file ijms-23-13697-s001.zip › ijms-2010141-supplementary.pdf]

International Journal of *Molecular Sciences*

MDPI

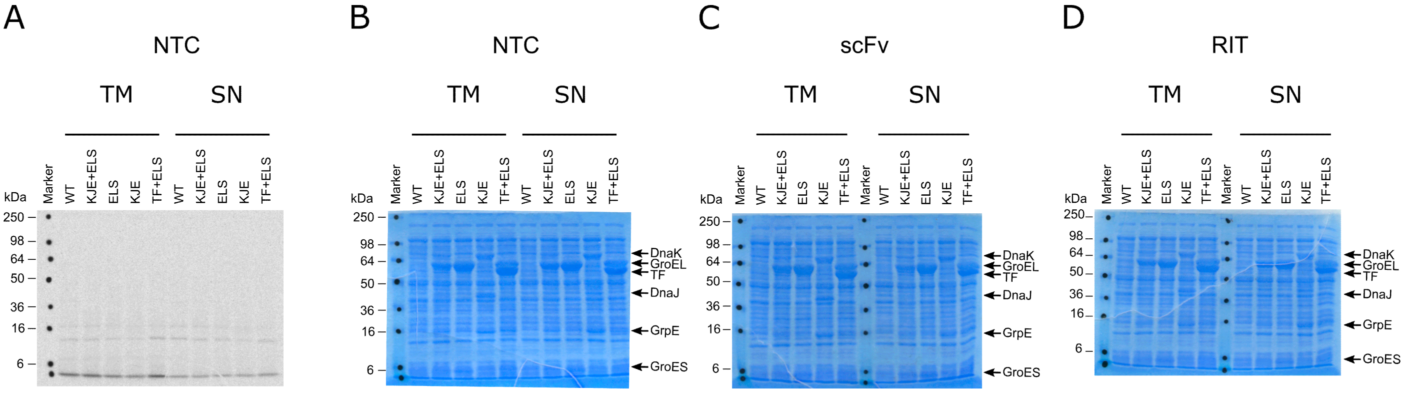

Figure S1. SDS-PAGE autoradiograph of NTC and blue gels of NTC, scFv and RIT (*E. coli* CFPS with and without chaperones). (**A**) Autoradiography of reducing, denatured 12 % Tris-Glycine SDS-PAGE blue gel of TM and SN fraction of NTC with (**B**) corresponding blue gel. (**C**) Reducing, denatured 12 % Tris-Glycine SDS-PAGE-blue gel of scFv (29.9 kDa) and (**D**) Reducing, denatured 12 % Tris-Glycine SDS-PAGE-blue gel of RIT (56.4 kDa) in TM and SN fraction. The chaperones' expected molecular weights are 70 kDa for DnaK, 40 kDa for DnaJ, 22 kDa for GrpE, 60 kDa for GroEL, 10 kDa for GroES and 56 kDa for Trigger Factor. **Abbreviations.** NTC: no template control; scFv: single chain variable fragment; RIT: recombinant immunotoxin; CFPS: cell-free protein synthesis; TM: translation mix; SN: supernatant; WT: Wild type BL21 Star™ (DE3) w/o chaperones; KJE+ELS: WT transformed with plasmid coding for DnaK/DnaJ/GrpE & GroEL/GroES; ELS: WT transformed with plasmid coding for GroEL/GroES; KJE: WT transformed with plasmid coding for DnaK/DnaJ/GrpE; TF+ELS: WT transformed with plasmid coding for Trigger Factor and GroEL/GroES.

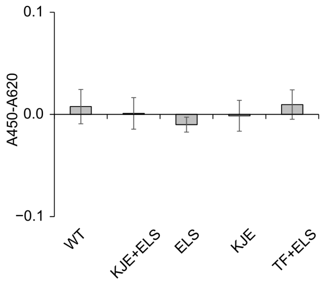

Figure S2. Indirect anti-CD7 ELISA of NTC (*E. coli* CFPS with and without chaperones). Soluble fraction (SN) of NTC from *E. coli* cell-free expression was diluted 1:20 and added to wells coated with CD7 and without CD7. Absorbance values in wells without CD7 were subtracted from absorbance values in wells coated with CD7. Error bars represent the standard deviation of triplicate analysis. **Abbreviations.** WT: Wild type BL21 Star™ (DE3) w/o chaperones; KJE+ELS: WT transformed with plasmid coding for DnaK/DnaJ/GrpE & GroEL/GroES; ELS: WT transformed with plasmid coding for GroEL/GroES; KJE: WT transformed with plasmid coding for DnaK/DnaJ/GrpE; TF+ELS: WT transformed with plasmid coding for Trigger Factor and GroEL/GroES.

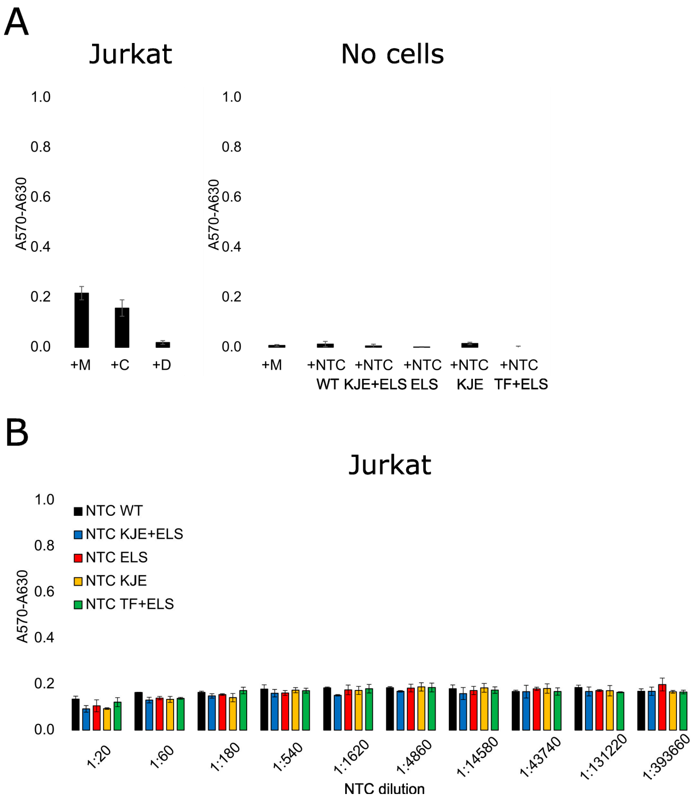

Figure S3. Influence of CHAPS, DDM and NTC on MTT assay signals (*E. coli* CFPS with and without chaperones) (**A**) MTT assay of Jurkat cells with addition of medium (+M), CHAPS (+C) and DDM (+D) (left) and medium (no cells) with addition of medium (+M) and addition of soluble fraction (SN) of 1:20 diluted NTCs (+NTC) from *E. coli* cell-free systems with or without chaperones (right). Error bars represent the standard deviation of triplicate analysis. (**B**) MTT assay of Jurkat cells with addition of NTC in dilutions from 1:20 to 1:393660 from *E. coli* cell-free systems with or without chaperones. **Abbreviations.** NTC: no template control; WT: Wild type BL21 Star™ (DE3) w/o chaperones; M: Medium; C: CHAPS (3-((3-cholamidopropyl) dimethylammonio)-1-propanesulfonate); D: DDM (n-Dodecyl-β-D-maltoside); NTC: no template control; WT: Wild type BL21 Star™ (DE3) w/o chaperones; KJE+ELS: WT transformed with plasmid coding for DnaK/DnaJ/GrpE & GroEL/GroES; ELS: WT transformed with plasmid coding for GroEL/GroES; KJE: WT transformed with plasmid coding for DnaK/DnaJ/GrpE; TF+ELS: WT transformed with plasmid coding for Trigger Factor and GroEL/GroES.

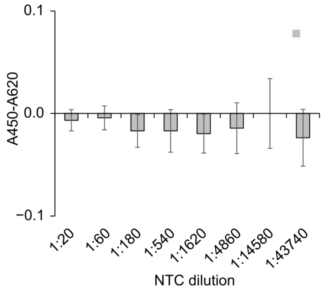

Figure S4. Indirect anti-CD7 ELISA of NTC (*E. coli* CFPS with GroES/GroEL). Soluble fraction (SN) of NTC from *E. coli* cell-free expression was diluted 1:20 to 1:43740 and added to wells coated with CD7 and without CD7. Absorbance values in wells without CD7 were subtracted from absorbance values in wells coated with CD7. Error bars represent the standard deviation of triplicate analysis.

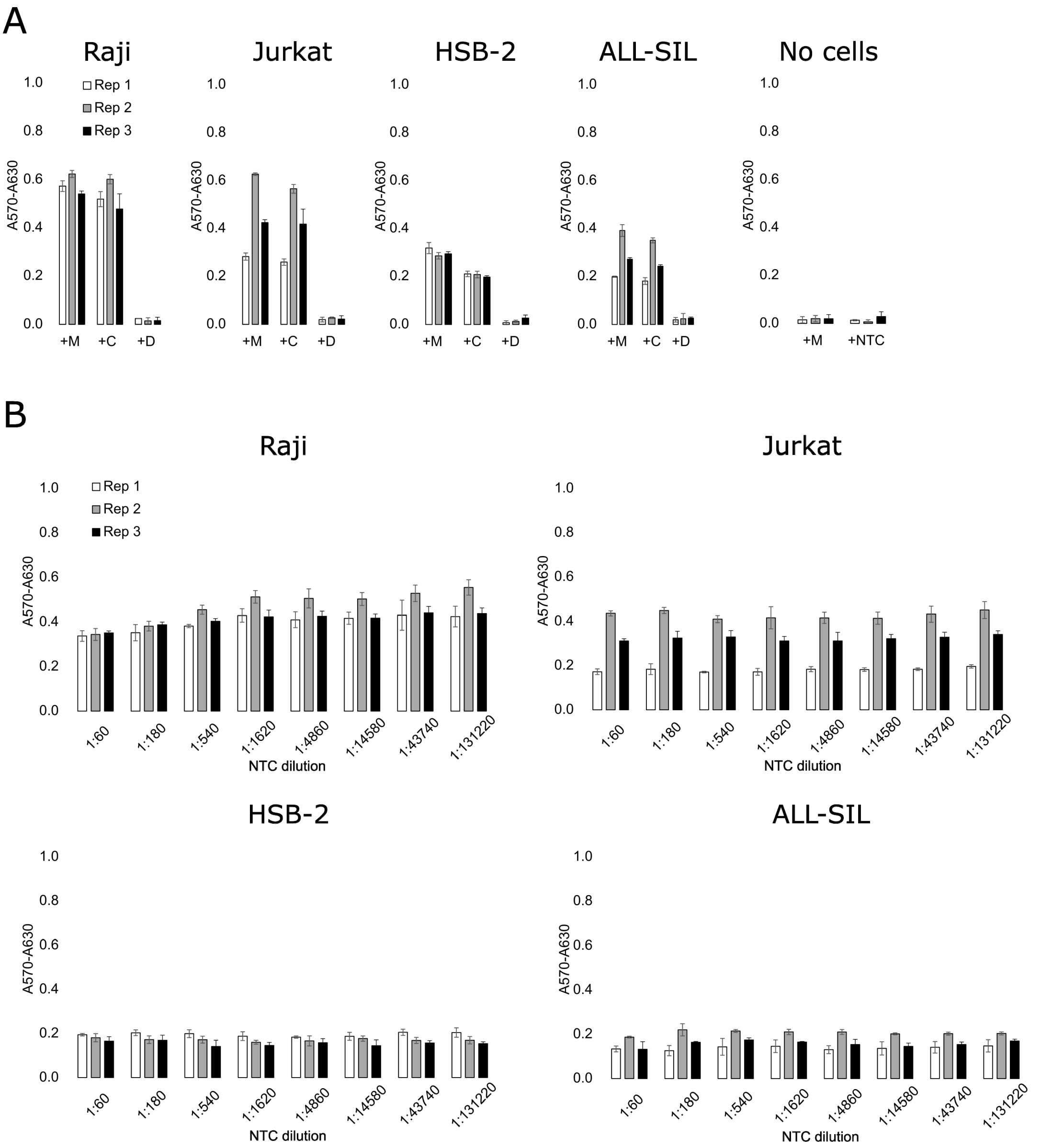

Figure S5. Influence of CHAPS, DDM and NTC on MTT assay signals (*E. coli* CFPS with GroEL/GroES). (**A**) MTT assay of three independent experiments (Rep1, Rep2, Rep3) of Raji, Jurkat, HSB-2 and ALL-SIL cells with addition of medium (+M), CHAPS (+C) and DDM (+D) (left) and medium (no cells) with addition of medium (+M) and addition of soluble fraction (SN) of 1:60 diluted NTC (+NTC, right). Error bars represent the standard deviation of triplicate analysis. (**B**) MTT assay of three independent experiments (Rep1, Rep2, Rep3) with Raji, Jurkat, HSB-2 and ALL-SIL cells with addition of NTC in dilutions from 1:60 to 1:131220 generated from *E. coli* cell-free system with GroES/GroEL chaperones. **Abbreviations.** Rep: Replicate; WT: Wild type BL21 Star™ (DE3) w/o chaperones; M: Medium; C: CHAPS (3-((3-cholamidopropyl) dimethylammonio)-1-propanesulfonate); D: DDM (n-Dodecyl-β-D-maltoside); NTC: no template control.

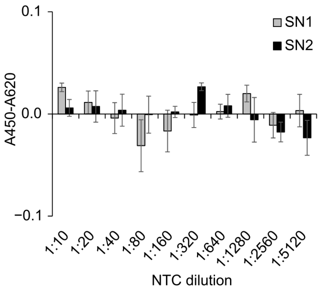

Figure S6. Indirect anti-CD7 ELISA of NTC (CHO CFPS). Soluble fractions SN1 and SN2 of NTC from CHO cell-free expression was diluted 1:10 to 1:5120 and added to wells coated with CD7 and without CD7. Absorbance values in wells without CD7 were subtracted from absorbance values in wells coated with CD7. Error bars represent the standard deviation of triplicate analysis. **Abbreviations.** NTC: no template control; SN: supernatant.

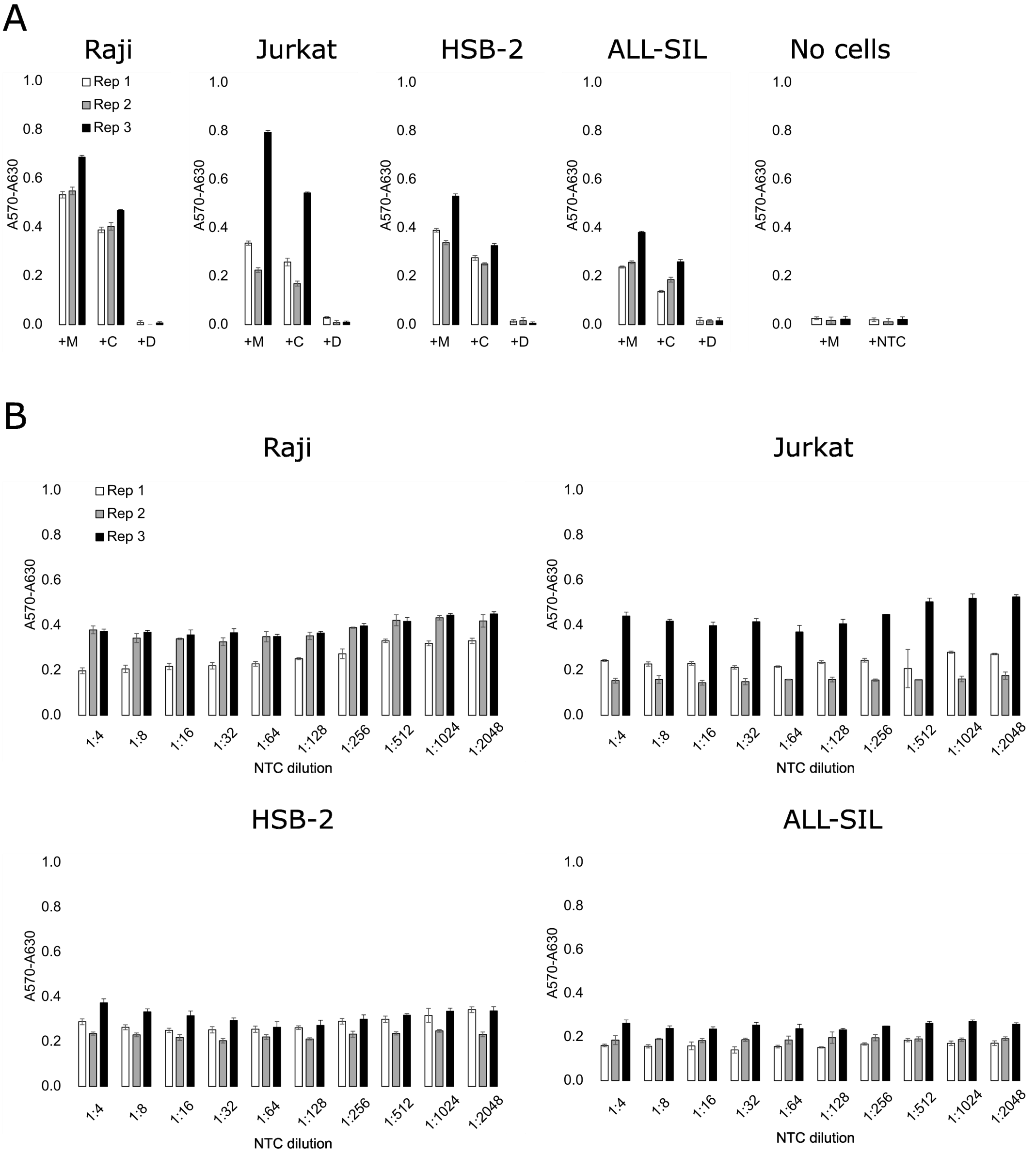

Figure S7: Influence of CHAPS, DDM and NTC on MTT assay signals (CHO CFPS). (A) MTT assay of three independent experiments (Rep1, Rep2, Rep3) of Raji, Jurkat, HSB-2 and ALL-SIL cells with addition of medium (+M), CHAPS (+C) and DDM (+D) (left) and medium (no cells) with addition of medium (+M) and addition of 1:4 diluted NTC of SN2 (+NTC, right). Error bars represent the

standard deviation of triplicate analysis. **(B)** MTT assay of three independent experiments (Rep1, Rep2, Rep3) with Raji, Jurkat, HSB-2 and ALL-SIL cells with addition of NTC of SN2 in dilutions from 1:4 to 1:2048 from CHO cell-free system. **Abbreviations.** NTC: no template control; Rep: Replicate; M: Medium; C: CHAPS (3-((3-cholamidopropyl) dimethylammonio)-1-propanesulfonate); D: DDM (n-Dodecyl-β-D-maltoside).

A

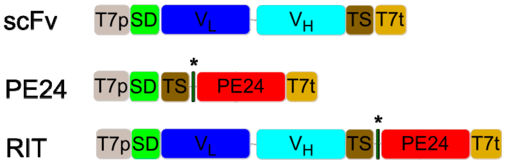

B

>puC57-1.8k_Ec-scFv-TS (scFv plasmid template for *E. coli* CFPS)

GATATGATATACGTACGATAGGCTAGCTAATACGACTCACTATAGGGAGACCACAACGGTTTCCCTCTAGAAATAATTTTGTTTAAC
TTTAAGAAGGAGATAAACATCCATGGGTGGTAGCGATATTCAGATGACCCAGACCACCAGTAGCCTGAGCGCAAGCCTGGGTGATCG
TGTTACCATTAGCTGTAGCGCCAGCCAGGGTATTAGCAATTATCTGAATTGGTATCAGCAGAAACCGGATGGCACCGTTAAACTGCT
GATCTATTATACCAGCAGCCTGCATAGCGGTGTTCCGAGCCGTTTTAGCGGTAGCGGTAGTGGCACCGATTATAGCCTGACCATTAG
CAATCTGGAACCGGAAGATATTGCCACCTATTATTGTCAGCAGTATAGCAAACTGCCGTATACCTTTGGTGGTGGCACCAAACTGGA
AATTAAACGCGGTGGTGGTGGTAGTGGTGGCGGTGGTTCAGGCGGTGGCGGTTCAGGTGGCGGAGGTAGCGAAGTTCAGCTGGTTGA
ATCAGGTGGTGGCCTGGTTAAACCTGGTGGTAGCCTGAAACTGAGCTGTGCAGCAAGCGGTCTGACCTTTAGCAGCTATGCAATGAG
CTGGGTTCGTCAGACACCGGAAAAACGTCTGGAATGGGTTGCAAGCATTAGCAGCGGTGGTTTTACCTATTATCCGGATTCAGTTAA
AGGTCGCTTTACCATTTCTCGTGATAATGCCCGTAATATCCTGTATCTGCAAATGAGCAGTCTGCGTAGCGAAGATACCGCAATGTA
TTATTGTGCACGTGATGAAGTTCGTGGTTATCTGGATGTTTGGGGTGCAGGCACCACCGTTACCGTTAGCAGCGCAGCAGCCGGTGG
CTCAGGTGGTAGCAGCGCCTGGTCACATCCGCAGTTTGAAAAAGGTGGTGGATCCGGTGGCGGAAGCGGTGGTAGCTCAGCTTGGAG
CCATCCTCAGTTCGAGAAA**TAA**TAACTAACTAACCA**Ag**ATCTgTACCCCTTggggCCTCTAAACgggTCTTgAggggTTTTTTggAT
CCgAATTCACCggTGATATCATATCACATGTGAGCAAAAGGCCAGCAAAAGGCCAGGAACCGTAAAAAGGCCGCGTTGCTGGCGTTT
TTCCATAGGCTCCGCCCCCCTGACGAGCATCACAAAAATCGACGCTCAAGTCAGAGGTGGCGAAACCCGACAGGACTATAAAGATAC
CAGGCGTTTCCCCCTGGAAGCTCCCTCGTGCGCTCTCCTGTTCCGACCCTGCCGCTTACCGGATACCTGTCCGCCTTTCTCCCTTCG
GGAAGCGTGGCGCTTTCTCATAGCTCACGCTGTAGGTATCTCAGTTCGGTGTAGGTCGTTCGCTCCAAGCTGGGCTGTGTGCACGAA
CCCCCCGTTCAGCCCGACCGCTGCGCCTTATCCGGTAACTATCGTCTTGAGTCCAACCCGGTAAGACACGACTTATCGCCACTGGCA
GCAGCCACTGGTAACAGGATTAGCAGAGCGAGGTATGTAGGCGGTGCTACAGAGTTCTTGAAGTGGTGGCCTAACTACGGCTACACT
AGAAGAACAGTATTTGGTATCTGCGCTCTGCTGAAGCCAGTTACCTTCGGAAAAAGAGTTGGTAGCTCTTGATCCGGCAAACAAACC
ACCGCTGGTAGCGGTGGTTTTTTTGTTTGCAAGCAGCAGATTACGCGCAGAAAAAAAGGATCTCAAGAAGATCCTTTGATCTTTTCT
ACGGGGTCTGACGCTCAGTGGAACGAAAACTCACGTTAAGGGATTTTGGTCATGAGATTATCAAAAAGGATCTTCACCTAGATCCTT
TTAAATTAAAAATGAAGTTTTAAATCAATCTAAAGTATATATGAGTAAACTTGGTCTGACAGTTACCAATGCTTAATCAGTGAGGCA
CCTATCTCAGCGATCTGTCTATTTCGTTCATCCATAGTTGCCTGACTCCCCGTCGTGTAGATAACTACGATACGGGAGGGCTTACCA
TCTGGCCCCAGTGCTGCAATGATACCGCGAGACCCACGCTCACCGGCTCCAGATTTATCAGCAATAAACCAGCCAGCCGGAAGGGCC
GAGCGCAGAAGTGGTCCTGCAACTTTATCCGCCTCCATCCAGTCTATTAATTGTTGCCGGGAAGCTAGAGTAAGTAGTTCGCCAGTT
AATAGTTTGCGCAACGTTGTTGCCATTGCTACAGGCATCGTGGTGTCACGCTCGTCGTTTGGTATGGCTTCATTCAGCTCCGGTTCC
CAACGATCAAGGCGAGTTACATGATCCCCCATGTTGTGCAAAAAAGCGGTTAGCTCCTTCGGTCCTCCGATCGTTGTCAGAAGTAAG
TTGGCCGCAGTGTTATCACTCATGGTTATGGCAGCACTGCATAATTCTCTTACTGTCATGCCATCCGTAAGATGCTTTTCTGTGACT

GGTGAGTACTCAACCAAGTCATTCTGAGAATAGTGTATGCGGCGACCGAGTTGCTCTTGCCCGGCGTCAATACGGGATAATACCGCG
CCACATAGCAGAACTTTAAAAGTGCTCATCATTGGAAAACGTTCTTCGGGGCGAAAACTCTCAAGGATCTTACCGCTGTTGAGATCC
AGTTCGATGTAACCCACTCGTGCACCCAACTGATCTTCAGCATCTTTTACTTTCACCAGCGTTTCTGGGTGAGCAAAAACAGGAAGG
CAAAATGCCGCAAAAAAGGGAATAAGGGCGACACGGAAATGTTGAATACTCATACTCTTCCTTTTTCAATATTATTGAAGCATTTAT
CAGGGTTATTGTCTCATGAGCGGATACATATTTGAATGTATTTAGAAAAATAAACAAATAGGGGTTCCGCGCACATTTCCCCGAAAA
GTGCCACCTGACGTC

>Ec-TS-PE24KDEL (PE24 PCR template for *E. coli* CFPS)

ATGATATACGTACGATAGGCTAGCTAATACGACTCACTATAGGGAGACCACAACGGTTTCCCTCTAGAAATAATTTTGTTTAACTTT
AAGAAGGAGATAAACATCCATGGGTGGTAGCAGCGCATGGTCACATCCGCAGTTTGAAAAAGGTGGTGGTAGCGGTGGTGGTTCAGG
TGGTAGCTCTGCTTGGAGCCATCCTCAGTTCGAGAAAGGCGGTAGTGGTGGCAGCCGTCATCGTCAGCCTCGTGGTTGGGAACAGCT
GCCGACCGGTGCAGAATTTTTAGGTGATGGTGGTGATATTAGCTTTAGCACCCGTGGCACCCAGAATTGGACCGTTGAACGTCTGCT
GCAGGCACACCGTCAGCTGGAAGAACGTGGTTATGTTTTTGTTGGTTATCATGGCACCTTTCTGGAAGCAGCACAGAGCATTGTTTT
TGGTGGTGTTCGTGCACGTAGCCAGGATCTGGATGCAATTTGGCGTGGTTTCTATATTGCCGGTGATCCGGCACTGGCATATGGTTA
TGCACAGGATCAAGAACCGGATGCACGTGGTCGTATTCGTAATGGTGCACTGCTGCGTGTTTATGTTCCGCGTAGCAGCCTGCCTGG
TTTTTATCGTACCGGTCTGACCCTGGCAGCACCGGAAGCAGCCGGTGAAGTGGAACGTCTGATTGGTCATCCGCTGCCGCTGCGTCT
GGATGCCATTACCGGTCCGGAAGAAGAAGGCGGTCGTCTGGAAACCATTTTAGGTTGGCCTCTGGCAGAACGTACCGTTGTTATTCC
GAGCGCAATTCCGACCGATCCGCGTAATGTTGGTGGCGATCTGGATCCGAGCAGCATTCCGGATAAAGAACAGGCAATTAGCGCACT
GCCGGATTATGCAAGCCAGCCTGGTAAACCGCCTAAAGATGAACTGTAATAACTAACTAACCAAgATCTgTACCCCTTggggCCTCT
AAACgggTCTTgAggggT

>PCR_Ec-scFv-TS-PE24KDEL (RIT PCR template for *E. coli* CFPS)

ATGATATACGTACGATAGGCTAGCTAATACGACTCACTATAGGGAGACCACAACGGTTTCCCTCTAGAAATAATTTTGTTTAACTTT
AAGAAGGAGATAAACATCCATGGGTGGTAGCGATATTCAGATGACCCAGACCACCAGTAGCCTGAGCGCAAGCCTGGGTGATCGTGT
TACCATTAGCTGTAGCGCCAGCCAGGGTATTAGCAATTATCTGAATTGGTATCAGCAGAAACCGGATGGCACCGTTAAACTGCTGAT
CTATTATACCAGCAGCCTGCATAGCGGTGTTCCGAGCCGTTTTAGCGGTAGCGGTAGTGGCACCGATTATAGCCTGACCATTAGCAA
TCTGGAACCGGAAGATATTGCCACCTATTATTGTCAGCAGTATAGCAAACTGCCGTATACCTTTGGTGGTGGCACCAAACTGGAAAT
TAAACGCGGTGGTGGTGGTAGTGGTGGCGGTGGTTCAGGCGGTGGCGGTTCAGGTGGCGGAGGTAGCGAAGTTCAGCTGGTTGAATC
AGGTGGTGGCCTGGTTAAACCTGGTGGTAGCCTGAAACTGAGCTGTGCAGCAAGCGGTCTGACCTTTAGCAGCTATGCAATGAGCTG
GGTTCGTCAGACACCGGAAAAACGTCTGGAATGGGTTGCAAGCATTAGCAGCGGTGGTTTTACCTATTATCCGGATTCAGTTAAAGG
TCGCTTTACCATTTCTCGTGATAATGCCCGTAATATCCTGTATCTGCAAATGAGCAGTCTGCGTAGCGAAGATACCGCAATGTATTA
TTGTGCACGTGATGAAGTTCGTGGTTATCTGGATGTTTGGGGTGCAGGCACCACCGTTACCGTTAGCAGCGCAGCAGCCGGTGGCTC
AGGTGGTAGCAGCGCCTGGTCACATCCGCAGTTTGAAAAAGGTGGTGGATCCGGTGGCGGAAGCGGTGGTAGCTCAGCTTGGAGCCA
TCCTCAGTTCGAGAAAGGTAGCTTAGGTAGCGGTGGCTCTGGTTCTGGTAGTGGCGGTAGTTTAGGTGGTAGTAGCCGTCATCGTCA
GCCTCGTGGTTGGGAACAGCTGCCGACCGGTGCAGAATTTTTAGGTGATGGTGGTGATATTAGCTTTAGCACCCGTGGCACCCAGAA
TTGGACCGTTGAACGTCTGCTGCAGGCACACCGTCAGCTGGAAGAACGTGGTTATGTTTTTGTTGGTTATCATGGCACCTTTCTGGA
AGCAGCACAGAGCATTGTTTTTGGTGGTGTTCGTGCACGTAGCCAGGATCTGGATGCAATTTGGCGTGGTTTCTATATTGCCGGTGA
TCCGGCACTGGCATATGGTTATGCACAGGATCAAGAACCGGATGCACGTGGTCGTATTCGTAATGGTGCACTGCTGCGTGTTTATGT
TCCGCGTAGCAGCCTGCCTGGTTTTTATCGTACCGGTCTGACCCTGGCAGCACCGGAAGCAGCCGGTGAAGTGGAACGTCTGATTGG
TCATCCGCTGCCGCTGCGTCTGGATGCCATTACCGGTCCGGAAGAAGAAGGCGGTCGTCTGGAAACCATTTTAGGTTGGCCTCTGGC
AGAACGTACCGTTGTTATTCCGAGCGCAATTCCGACCGATCCGCGTAATGTTGGTGGCGATCTGGATCCGAGCAGCATTCCGGATAA

AGAACAGGCAATTAGCGCACTGCCGGATTATGCAAGCCAGCCTGGTAAACCGCCTAAAGATGAACTG**TAA**TAACTAACTAACCAAgA
TCTgTACCCCTTggggCCTCTAAACgggTCTTgAggggT

Annotation

Restriction sites: *NheI*, *NcoI*, *BglII*

Regulatory elements: T7 promotor, Shine-Dalgarno sequence, **stop codon**, T7 terminator

Coding sequence: Melittin signal peptide, V_L region, V_L-V_H linker, V_H region, Twin Strep tag, **Furin cleavage site**, PE24

Figure S8: Schematics and sequences of DNA templates used for *E. coli* CFPS. (A) Schematics of DNA templates used for *E. coli* cell-free protein synthesis. The furin cleavage site upstream of PE24 is marked by an asterisk. **(B)** DNA Sequence of scFv in pUC57-1.8k and PCR template of PE24 and scFv-PE24 RIT. **Abbreviations.** scFv: single chain variable fragment; PE24: 24 kDa variant of Pseudomonas Exotoxin A; RIT: recombinant immunotoxin; T7p: T7 polymerase promotor; SD: Shine-Dalgarno sequence; V_L: variable light chain; V_H: variable heavy chain; TS: Twin Strep Tag; PE24: 24 kDa variant of Pseudomonas Exotoxin A; T7t: T7 polymerase terminator.

A

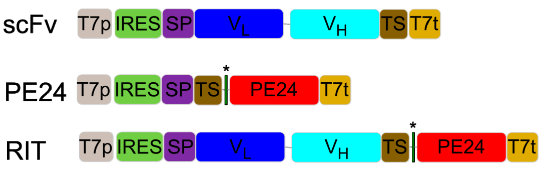

B

>puC57-1.8k_CHO-scFv-TS (scFv plasmid template for CHO CFPS)

GATATGATATCTCGAGCGGCCGCTAGCTAATACGACTCACTATAGGGAGACCACAACGGTTTCCCTCTAGAAATAATTTTGTTTAAC
TTTAAGAAGGAGATAAACAAAAGCAAAAATGTGATCTTGCTTGTAAATACAATTTTGAGAGGTTAATAAATTACAAGTAGTGCTATT
TTTGTATTTAGGTTAGCTATTTAGCTTTACGTTCCAGGATGCCTAGTGGCAGCCCCACAATATCCAGGAAGCCCTCTCTGCGGTTTT
TCAGATTAGGTAGTCGAAAAACCTAAGAAATTTACCTGCTAAATTCTTAGTCAACGTTGCCCTTGTTTTTATGGTCGTATACATTTC
TTACATCTATGCGGACATCCAGATGACCCAGACCACCTCCAGCCTGTCTGCTTCTCTGGGCGACAGAGTGACCATCAGTTGTAGCGC
ATCCCAGGGCATCTCCAACTACCTGAACTGGTATCAGCAGAAACCCGACGGCACCGTGAAGCTGCTGATCTACTACACCAGCAGCCT
GCACTCTGGCGTGCCCTCTAGATTTTCCGGCTCTGGCTCTGGCACCGACTACTCCCTGACCATCAGCAACCTGGAACCTGAGGATAT
CGCCACCTACTACTGCCAGCAGTACTCCAAGCTGCCCTACACCTTTGGCGGAGGCACCAAGCTGGAAATCAAGAGAGGTGGCGGAGG
TTCTGGCGGTGGAGGTTCTGGCGGAGGCGGAAGTGGCGGTGGAGGTAGTGAAGTTCAGCTGGTTGAATCAGGCGGAGGCCTGGTTAA
GCCTGGCGGATCTCTGAAGCTGTCTTGTGCCGCTTCTGGCCTGACCTTCTCCTCCTACGCTATGTCCTGGGTCCGACAGACCCCTGA
GAAGAGACTGGAATGGGTCGCCTCCATCTCCTCCGGCGGCTTCACCTACTATCCCGACTCTGTGAAGGGCAGATTCACCATCTCTCG
GGACAACGCCCGGAACATCCTGTACCTGCAGATGTCCAGCCTGCGGAGCGAGGATACCGCTATGTACTACTGCGCCAGGGATGAAGT
GCGGGGCTACCTGGATGTTTGGGGCGCTGGAACAACCGTGACCGTGTCATCTGGAGGAAGTGGCGGTTCCAGCGCATGGTCCCATCC
TCAGTTCGAGAAAGGCGGAGGATCTGGCGGAGGTAGCGGTGGATCTAGTGCTTGGAGCCACCCCCAGTTTGAAAAG**TAA**TAA**CTAAC**

TAACCAAgATCTgTACCCCTTggggCCTCTAAACgggTCTTgAggggTTTTTTggATCCgAATTCACCggTGATATCATATCACATG
TGAGCAAAAGGCCAGCAAAAGGCCAGGAACCGTAAAAAGGCCGCGTTGCTGGCGTTTTTCCATAGGCTCCGCCCCCCTGACGAGCAT
CACAAAAATCGACGCTCAAGTCAGAGGTGGCGAAACCCGACAGGACTATAAAGATACCAGGCGTTTCCCCCTGGAAGCTCCCTCGTG
CGCTCTCCTGTTCCGACCCTGCCGCTTACCGGATACCTGTCCGCCTTTCTCCCTTCGGGAAGCGTGGCGCTTTCTCATAGCTCACGC
TGTAGGTATCTCAGTTCGGTGTAGGTCGTTCGCTCCAAGCTGGGCTGTGTGCACGAACCCCCCGTTCAGCCCGACCGCTGCGCCTTA
TCCGGTAACTATCGTCTTGAGTCCAACCCGGTAAGACACGACTTATCGCCACTGGCAGCAGCCACTGGTAACAGGATTAGCAGAGCG
AGGTATGTAGGCGGTGCTACAGAGTTCTTGAAGTGGTGGCCTAACTACGGCTACACTAGAAGAACAGTATTTGGTATCTGCGCTCTG
CTGAAGCCAGTTACCTTCGGAAAAAGAGTTGGTAGCTCTTGATCCGGCAAACAAACCACCGCTGGTAGCGGTGGTTTTTTTGTTTGC
AAGCAGCAGATTACGCGCAGAAAAAAAGGATCTCAAGAAGATCCTTTGATCTTTTCTACGGGGTCTGACGCTCAGTGGAACGAAAAC
TCACGTTAAGGGATTTTGGTCATGAGATTATCAAAAAGGATCTTCACCTAGATCCTTTTAAATTAAAAATGAAGTTTTAAATCAATC
TAAAGTATATATGAGTAAACTTGGTCTGACAGTTACCAATGCTTAATCAGTGAGGCACCTATCTCAGCGATCTGTCTATTTCGTTCA
TCCATAGTTGCCTGACTCCCCGTCGTGTAGATAACTACGATACGGGAGGGCTTACCATCTGGCCCCAGTGCTGCAATGATACCGCGA
GACCCACGCTCACCGGCTCCAGATTTATCAGCAATAAACCAGCCAGCCGGAAGGGCCGAGCGCAGAAGTGGTCCTGCAACTTTATCC
GCCTCCATCCAGTCTATTAATTGTTGCCGGGAAGCTAGAGTAAGTAGTTCGCCAGTTAATAGTTTGCGCAACGTTGTTGCCATTGCT
ACAGGCATCGTGGTGTCACGCTCGTCGTTTGGTATGGCTTCATTCAGCTCCGGTTCCCAACGATCAAGGCGAGTTACATGATCCCCC
ATGTTGTGCAAAAAAGCGGTTAGCTCCTTCGGTCCTCCGATCGTTGTCAGAAGTAAGTTGGCCGCAGTGTTATCACTCATGGTTATG
GCAGCACTGCATAATTCTCTTACTGTCATGCCATCCGTAAGATGCTTTTCTGTGACTGGTGAGTACTCAACCAAGTCATTCTGAGAA
TAGTGTATGCGGCGACCGAGTTGCTCTTGCCCGGCGTCAATACGGGATAATACCGCGCCACATAGCAGAACTTTAAAAGTGCTCATC
ATTGGAAAACGTTCTTCGGGGCGAAAACTCTCAAGGATCTTACCGCTGTTGAGATCCAGTTCGATGTAACCCACTCGTGCACCCAAC
TGATCTTCAGCATCTTTTACTTTCACCAGCGTTTCTGGGTGAGCAAAAACAGGAAGGCAAAATGCCGCAAAAAAGGGAATAAGGGCG
ACACGGAAATGTTGAATACTCATACTCTTCCTTTTTCAATATTATTGAAGCATTTATCAGGGTTATTGTCTCATGAGCGGATACATA
TTTGAATGTATTTAGAAAAATAAACAAATAGGGGTTCCGCGCACATTTCCCCGAAAAGTGCCACCTGACGTC

>CHO-TS-PE24KDEL (PE24 PCR template for CHO CFPS)

ATGATATACGTACGATAG**GCTAGC**TAATACGACTCACTATAGGGAGACCACAACGGTTTCCCTCTAGAAATAATTTTGTTTAACTTT
AAGAAGGAGATAAACAAAAGCAAAAATGTGATCTTGCTTGTAAATACAATTTTGAGAGGTTAATAAATTACAAGTAGTGCTATTTTT
GTATTTAGGTTAGCTATTTAGCTTTACGTTCCAGGATGCCTAGTGGCAGCCCCACAATATCCAGGAAGCCCTCTCTGCGGTTTTTCA
GATTAGGTAGTCGAAAAACCTAAGAAATTTACCT**GCT**AAATTCTTAGTCAACGTTGCCCTTGTTTTTATGGTC**GTATAC**ATTTCTTA
CATCTATGCGAGCGCATGGTCCCATCCTCAGTTCGAGAAAGGCGGAGGATCTGGCGGAGGTAGCGGTGGATCTAGTGCTTGGAGCCA
CCCCCAGTTTGAAAAGGGAGGAAGTGGCGGTTCC**AGACACCGGCAGCCTAGAGGATGGGAGCAGCTG**CCTACCGGCGCTGAGTTCCT
CGGAGATGGCGGCGACATCTCTTTCAGCACCAGAGGCACCCAGAACTGGACCGTGGAAAGACTGCTGCAGGCCCACAGACAGCTCGA
AGAGCGGGGCTATGTGTTCGTGGGCTACCACGGCACCTTTCTGGAAGCCGCTCAGTCCATCGTGTTCGGCGGAGTCAGAGCTAGATC
CCAGGACCTGGACGCCATTTGGCGGGGCTTTTACATTGCTGGCGATCCCGCTCTGGCCTACGGCTACGCTCAAGACCAAGAGCCTGA
CGCCAGGGGCAGAATCAGAAATGGCGCTCTGCTGAGAGTGTACGTGCCCAGAAGTTCCCTGCCAGGCTTCTACAGAACCGGACTGAC
TCTGGCCGCTCCTGAAGCAGCTGGCGAGGTGGAAAGGCTGATCGGACATCCTCTGCCACTGCGGCTGGATGCTATCACCGGACCTGA
AGAAGAAGGCGGACGGCTGGAAACCATCCTCGGATGGCCTCTGGCCGAGAGAACAGTGGTCATCCCCAGCGCTATCCCTACCGATCC
TAGAAACGTCGGCGGCGATCTGGACCCCTCTAGCATCCCTGACAAAGAGCAGGCCATCTCTGCCCTGCCTGACTACGCTTCTCAGCC
TGGCAAACCTCCTAAAGATGAGCTG**TAA**TAACTAACTAACCAAgATCTgTACCCCTTggggCCTCTAAACgggTCTTgAggggT

>CHO-scFv-TS-PE24KDEL (RIT PCR template for CHO CFPS)

ATGATATACGTACGATAGGCTAGCTAATACGACTCACTATAGGGAGACCACAACGGTTTCCCTCTAGAAATAATTTTGTTTAACTTT
AAGAAGGAGATAAACAAAAGCAAAAATGTGATCTTGCTTGTAAATACAATTTTGAGAGGTTAATAAATTACAAGTAGTGCTATTTTT
GTATTTAGGTTAGCTATTTAGCTTTACGTTCCAGGATGCCTAGTGGCAGCCCCACAATATCCAGGAAGCCCTCTCTGCGGTTTTTCA
GATTAGGTAGTCGAAAAACCTAAGAAATTTACCTGCTAAATTCTTAGTCAACGTTGCCCTTGTTTTTATGGTCGTATACATTTCTTA
CATCTATGCGTCCATGGGAGGTTCAGACATCCAGATGACCCAGACCACCTCCAGCCTGTCTGCTTCTCTGGGCGACAGAGTGACCAT
CAGTTGTAGCGCATCCCAGGGCATCTCCAACTACCTGAACTGGTATCAGCAGAAACCCGACGGCACCGTGAAGCTGCTGATCTACTA
CACCAGCAGCCTGCACTCTGGCGTGCCCTCTAGATTTTCCGGCTCTGGCTCTGGCACCGACTACTCCCTGACCATCAGCAACCTGGA
ACCTGAGGATATCGCCACCTACTACTGCCAGCAGTACTCCAAGCTGCCCTACACCTTTGGCGGAGGCACCAAGCTGGAAATCAAGAG
AGGTGGCGGAGGTTCTGGCGGTGGAGGTTCTGGCGGAGGCGGAAGTGGCGGTGGAGGTAGTGAAGTTCAGCTGGTTGAATCAGGCGG
AGGCCTGGTTAAGCCTGGCGGATCTCTGAAGCTGTCTTGTGCCGCTTCTGGCCTGACCTTCTCCTCCTACGCTATGTCCTGGGTCCG
ACAGACCCCTGAGAAGAGACTGGAATGGGTCGCCTCCATCTCCTCCGGCGGCTTCACCTACTATCCCGACTCTGTGAAGGGCAGATT
CACCATCTCTCGGGACAACGCCCGGAACATCCTGTACCTGCAGATGTCCAGCCTGCGGAGCGAGGATACCGCTATGTACTACTGCGC
CAGGGATGAAGTGCGGGGCTACCTGGATGTTTGGGGCGCTGGAACAACCGTGACCGTGTCATCTGCGGCCGCAGGAGGAAGTGGCGG
TTCCAGCGCATGGTCCCATCCTCAGTTCGAGAAAGGCGGAGGATCTGGCGGAGGTAGCGGTGGATCTAGTGCTTGGAGCCACCCCCA
GTTTGAAAAGGGAAGCTTGGGAAGTGGTGGATCTGGCTCTGGAAGTGGAGGAAGTTTAGGAGGCTCGAGT**AGACACCGGCAGCCTAG**
AGGATGGGAGCAGCTGCCTACCGGCGCTGAGTTCCTCGGAGATGGCGGCGACATCTCTTTCAGCACCAGAGGCACCCAGAACTGGAC
CGTGGAAAGACTGCTGCAGGCCCACAGACAGCTCGAAGAGCGGGGCTATGTGTTCGTGGGCTACCACGGCACCTTTCTGGAAGCCGC
TCAGTCCATCGTGTTCGGCGGAGTCAGAGCTAGATCCCAGGACCTGGACGCCATTTGGCGGGGCTTTTACATTGCTGGCGATCCCGC
TCTGGCCTACGGCTACGCTCAAGACCAAGAGCCTGACGCCAGGGGCAGAATCAGAAATGGCGCTCTGCTGAGAGTGTACGTGCCCAG
AAGTTCCCTGCCAGGCTTCTACAGAACCGGACTGACTCTGGCCGCTCCTGAAGCAGCTGGCGAGGTGGAAAGGCTGATCGGACATCC
TCTGCCACTGCGGCTGGATGCTATCACCGGACCTGAAGAAGAAGGCGGACGGCTGGAAACCATCCTCGGATGGCCTCTGGCCGAGAG
AACAGTGGTCATCCCCAGCGCTATCCCTACCGATCCTAGAAACGTCGGCGGCGATCTGGACCCCTCTAGCATCCCTGACAAAGAGCA
GGCCATCTCTGCCCTGCCTGACTACGCTTCTCAGCCTGGCAAACCTCCTAAAGATGAGCTG**TAA**TAACTAACTAACCAAgATCTgTA
CCCCTTggggCCTCTAAACgggTCTTgAggggT

Annotation

Restriction sites: *XhoI*, *NotI*, *NheI*, *BglII*, *BamHI*, *EcoRI*, *AgeI*, *NcoI*, *HindIII*

Regulatory elements: T7 promotor, IRES, **stop codon**, T7 terminator

Coding sequence: Melittin signal peptide, V_L region, V_L-V_H linker, V_H region, Twin Strep tag, **Furin cleavage site**, PE24

Figure S9: Schematics and sequences of DNA templates used for CHO CFPS. (A) Schematics of DNA template design for CHO cell-free protein synthesis. The furin cleavage site upstream of PE24 is marked by an asterisk. **(B)** DNA Sequence of scFv in pUC57-1.8k and PCR template of PE24 and scFv-PE24 RIT. **Abbreviations.** scFv: single chain variable fragment; PE24: 24 kDa variant of Pseudomonas Exotoxin A; RIT: recombinant immunotoxin; T7p: T7 polymerase promotor; IRES: internal ribosomal entry site from Cricket paralysis virus; SP: Melittin signal peptide; V_L: variable light chain; V_H: variable heavy chain; TS: Twin Strep Tag; T7t: T7 polymerase terminator.
